# Inhibiting Bacterioferritin Iron Release Induces Iron Starvation and Metabolic Downshift, Potentiating Aminoglycosides in *P. aeruginosa* Biofilms

**DOI:** 10.1021/acsinfecdis.6c00329

**Published:** 2026-06-15

**Authors:** Leo Fontenot, Huili Yao, Alexanndra M. Behm, Anabel Soldano, Fabrizio Donnarumma, Richard A. Bunce, Mario Rivera

**Affiliations:** † Department of Chemistry, 5779 Louisiana State University, Baton Rouge, Louisiana 70803, United States; ‡ Department of Chemistry, 7618 Oklahoma State University, Stillwater, Oklahoma 74078, United States

**Keywords:** antibiotic, biofilm, iron homeostasis, bacterioferritin, iron storage, combination treatment, Pseudomonas aeruginosa

## Abstract

In *Pseudomonas aeruginosa*, bacterioferritin contributes to regulating cytosolic iron by oxidizing Fe^2+^ and storing Fe^3+^ within its internal cavity, and binding to its cognate ferredoxin to reduce stored Fe^3+^ and release Fe^2+^ to the cytosol. Small molecule derivatives of 4-aminoisoindoline-1,3-dione bind *P. aeruginosa* bacterioferritin at the cognate ferredoxin binding site, inhibit iron release from bacterioferritin, and exhibit bactericidal activity against mature *P. aeruginosa* biofilms. In this study we report that treatment of mature biofilms with the 4-aminoisoindoline-1,3-dione derivative KM-5-35 induced approximately 90% cell death. Proteomic analysis of surviving cells revealed iron limitation accompanied by disrupted central carbon metabolism, reduced fatty acid and amino acid biosynthesis, and alterations in ribosome protein composition. These metabolic downshifts likely resulted in unstable proton motive force, diminished efflux capacity and impaired ribosome turnover. In this compromised state, gentamicin and amikacin, but not tobramycin, efficiently exploit KM-5-35-induced translational stress, leading to profound synergistic killing of biofilm cells. These findings underscore the therapeutic potential of targeting bacterioferritin iron mobilization in biofilm-associated infections and suggest a promising strategy for combination antimicrobial therapy.

## Graphical abstract

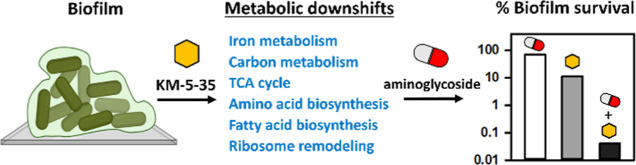

In 2018, the World Health Organization (WHO) identified carbapenem-resistant *Acinetobacter baumannii*, *Pseudomonas aeruginosa* and Enterobacterales as organisms for which new antibiotics are critically needed.^1^ Infections caused by these bacteria are difficult to treat and are associated with high morbidity and mortality.^2^ *P. aeruginosa*, a leading cause of hospital-acquired infections, is particularly adept at forming biofilms and colonizing wounds, endotracheal tubes, urinary catheters,^3^ as well as the lungs of cystic fibrosis patients.^4^ The prevalence of resistant *P. aeruginosa* infections continues to rise globally, contributing to high mortality, morbidity, and health care costs.^5,6^ This situation underscores the pressing need for new antibiotics and novel therapeutic targets.

One promising vulnerability is bacterial iron homeostasis. Host-mediated iron restriction reduces free iron levels to approximately 10^–18^ M,^7,8^ creating a challenging environment for bacteria, which typically require levels of free iron several orders of magnitude higher (10^–5^ to 10^–7^ M) for survival.^9,10^ Consequently, the ability of pathogens to survive and colonize the host depends on tightly regulated iron homeostasis, achieved through strategies that sense and respond to intracellular iron levels.^11^ When iron is limited, bacteria upregulate genes for iron acquisition, the mobilization of iron from iron storage proteins, and the subsequent incorporation of iron in proteins participating in bacterial cell metabolism.^11^ Our laboratories have been investigating bacterial iron storage proteins,^12–17^ leading to the pioneering concept of targeting the mobilization of iron from the bacterial storage protein bacterioferritin (Bfr) as a novel antibiotic strategy.^11^ Bfr, unique to bacteria, is a spherical, hollow protein capable of storing up to 2000 Fe^3+^ ions (Figure 1A). Bfr participates in the maintenance of iron homeostasis by catalyzing the oxidation of Fe^2+^ and storing the resultant Fe^3+^, thereby reducing Fe^2+^ participation in Fenton chemistry and allowing for high levels of Fe^3+^ storage, which would otherwise be insoluble at physiological pH.^11,12^ Our work has also overturned the long-held assumption that bacterioferritin and bacterial ferritins (Ftn) are always assembled from a single subunit type. We demonstrated that *P. aeruginosa* Bfr (*Pa* Bfr) and *A. baumannii* Bfr (*Ab* Bfr) are assembled from two types of homosubunit dimers, ferritin and the heme-binding bacterioferritin.^13,18^ The crystal structure of *Ab* Bfr (PDB 9BTS) revealed that the 24-mer is assembled from randomly distributed Bfr and Ftn subunit dimers, so the structure is described by an occupancy-weighted average of Bfr and Ftn subunit dimers.^18^

**Figure 1:**
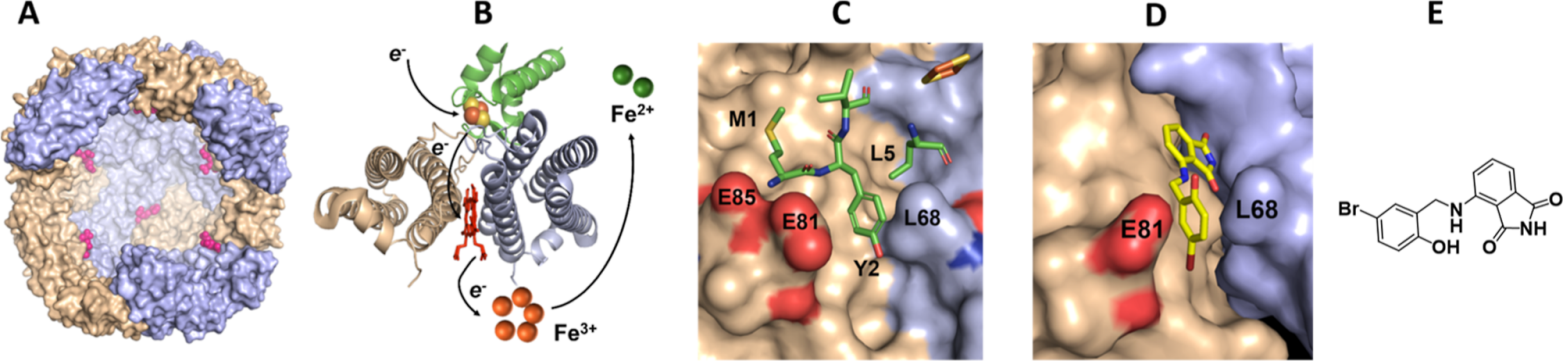
(A) Bfr from *P. aeruginosa* (PDB ID 3is7) is a spherical and hollow protein assembled from FtnA and BfrB homosubunit dimers, where heme binds between each BfrB homosubunit dimer. (B) Structure of the *P. aeruginosa* Bfr–Bfd complex (PDB ID 3E6K). Bfd (green) binds at the interface of a BfrB homosubunit dimer, above a heme molecule. Electron transfer from the [2Fe–2S] cluster in Bfd to the Fe^3+^ in Bfr facilitates mobilization of Fe^2+^. (C) Close-up view of the Bfr–Bfd interface showing Bfr residues in surface rendering and Bfd residues anchoring on the surface as sticks. (D) Crystal structure of KM-5-35 bound to the Bfd binding site on *P. aeruginosa* Bfr (PDB 9NHT). (E) The structure of KM-5-35.

Mobilization of stored iron from Bfr requires binding of its cognate ferredoxin (Bfd).^19–21^ Bfd binds Bfr at a specific site formed at the interface of a Bfr subunit dimer, at a shallow depression defined by residues L68, P69, N70, and Q72 from one subunit, and L78, L79, and G80 from the other, where Bfd residues M1, Y2, and L5 interact (Figure 1B,C). The Bfr–Bfd complex (*K*_d_ = 4.7 μM) enables electron transfer from the [2Fe–2S] cluster of Bfd to Fe^3+^ stored within the Bfr interior cavity via the heme, facilitating the release of Fe^2+^ to the cytosol. Hence, Bfr buffers cytosolic Fe^2+^ in *P. aeruginosa* by catalyzing the oxidation of Fe^2+^ and storing the resultant Fe^3+^, and by partnering with Bfd to release Fe^2+^ to the cytosol.^22^ Deletion of the *bfd* gene in *P. aeruginosa* disrupts iron homeostasis in planktonic cells, leading to an uncontrolled iron starvation response, metabolic dysregulation impacting carbon metabolism and amino acid biosynthesis,^23^ and an inability to mature biofilms regardless of environmental iron availability.^24^

Using a fragment based, structure-guided approach, we discovered novel 4-aminoisoindoline-1,3-dione derivatives that bind Bfr at the Bfd-binding site and inhibit iron mobilization. Crystal structures of Bfr bound to several of these derivatives revealed that the inhibitors consistently bind Bfr at the Bfd binding site, as illustrated by the structure of KM-5-35 bound to *Pa* Bfr^25–27^ (Figure 1D,E). These inhibitors penetrate the Gram-negative envelope, accumulate intracellularly, and exert a bactericidal effect against planktonic *A. baumannii* and biofilm-entrenched *P. aeruginosa*.^26,27^ In this study, we report that proteomic profiling *P. aeruginosa* biofilm cells that survive treatment with the Bfr–Bfd inhibitor KM-5-35 reveals evidence of iron starvation and metabolic downshift, impacting translation, carbon metabolism, the TCA cycle, and fatty acid and amino acid biosynthesis. Notably, the metabolic stress induced by KM-5-35 can be exploited by aminoglycosides to elicit deep killing of biofilm cells.

## Results and Discussion

In previous work, we developed a platform to study the susceptibility of mature *P. aeruginosa* biofilms to antimicrobials.^26,27^ To this end, 27 h biofilms were cultured at the air–liquid interface in PI media supplemented with 20 μM Fe. These mature biofilms were transferred onto glass coverslips by carefully touching the biofilm with the surface of the coverslip, followed by exposing the coverslip-adhered biofilms to the treatment solution consisting of AB media supplemented with 1.5% DMSO, 0.025% HPMC, 20 μM Fe and the antimicrobial agent under investigation. After treatment, biofilms were harvested, washed, and dispersed into sterile PBS by vortexing with zirconia beads prior to plating and enumerating viable cells (CFU/mL). These mature biofilms have demonstrated tolerance to tobramycin and ciprofloxacin at concentrations as high as 50× the MIC, but are susceptible to colistin at a concentration 25× MIC, and to inhibitors of the Bfr–Bfd complex in a concentration dependent manner.^26,27^ In this study, we treated similarly cultured *P. aeruginosa* biofilms with the Bfr–Bfd inhibitor KM-5-35, which induces concentration dependent biofilm cell death.^27^ Treatment with 50 μM KM-5-35 for 24 h resulted in approximately 10% cell survival relative to untreated controls (Figure 2A).

**Figure 2:**
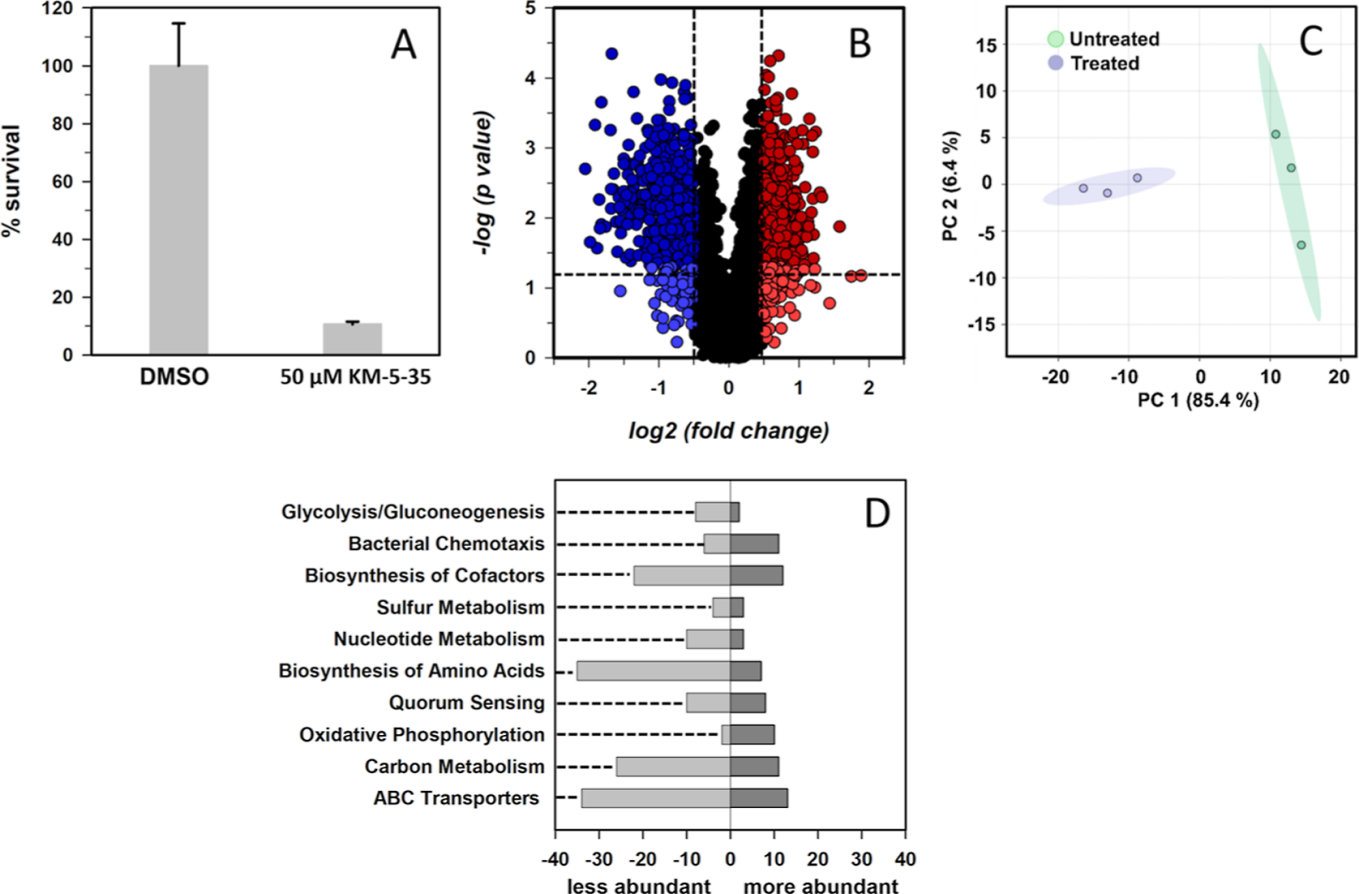
Effects of KM-5-35 on *P. aeruginosa* biofilms. (A) Treatment of *P. aeruginosa* biofilms with KM-5-35 for 24 h results in approximately 10% cell survival. Surviving cells were subjected to bottom-up proteomics. (B) Volcano plot illustrating differentially abundant proteins in KM-5-35-treated cells relative to untreated controls. The fold change (FC) is expressed as the abundance ratio, (treated/untreated). The horizontal dashed line denotes *p* = 0.05 and the vertical lines indicate log_2_ FC thresholds of ±0.5. (C) Principal component analysis plot. (D) KEGG Orthology-based functional classification of proteins with significant differential expression.

To compare the proteomic profile of the surviving biofilm cells and untreated controls, we performed Tandem Mass Tag (TMT) labeling, which enabled the identification of 3354 proteins, approximately 60% of the total proteins in *P. aeruginosa*.^28^ Three biological replicates were analyzed, and statistical significance was determined using *t*-tests (*p* < 0.05) to identify differences in protein abundance between untreated and KM-5-35-treated cells. Proteins exhibiting a treated/untreated fold change (FC) ≤ 0.7 (log_2_ FC ≤ −0.5) were classified as depleted, while those FC ≥ 1.4 (log_2_ FC ≥ 0.5) were considered enriched.^23^ These results are shown in Figure 2B, where the horizontal segmented line indicates *p* = 0.05 and the segmented vertical lines represent log_2_ FC ± 0.5. Of the 3354 identified proteins, 859 (∼26%) met the significance threshold (*p* < 0.05); among these, 416 (∼12%) were enriched, and 443 (∼13%) were depleted (Table S1) in the cells surviving KM-5-35 treatment compared to the untreated controls. Principal component analysis (PCA) (Figure 2C) revealed clear separation between treated and untreated groups, with each group forming distinct clusters and exhibiting high within-group homogeneity. Differentially expressed proteins were categorized using the Kyoto Encyclopedia of Genes and Genomes (KEGG) Orthology system (Figure 2D), the Pseudomonas Genome Database, and the UniProt database. Comparative untargeted LC–MS/MS cellular metabolomics analysis was performed to profile the surviving biofilm cells and the untreated control. The details are presented in the Experimental and the results summarized in Table S2.

### Evidence of Iron Limitation in Cells Treated with KM-5-35

We previously reported that *P. aeruginosa* stores iron in a heterooligomeric bacterioferritin (Bfr) assembled from bacterioferritin B (BfrB) and ferritin A (FtnA) homosubunit dimers.^13^ Bfr catalyzes the oxidation of Fe^2+^ and stores the mineralized Fe^3+^ in its hollow core.^12,29^ Mobilization of iron from Bfr to the cytosol requires binding and electron transfer from a ferredoxin (Bfd) to the Fe^3+^ mineral within Bfr, facilitating Fe^2+^ release (Figure 1B).^19,20,22^ Hence, the O_2_/H_2_O_2_-dependent Fe^2+^ oxidation and Fe^3+^ storage, and the Bfd-dependent mobilization of Fe^2+^ establishes a dynamic equilibrium that buffers cytosolic free iron at levels appropriate for Fur, the master regulator of iron homeostasis.^22^ Inhibition of the Bfr–Bfd complex, either by *bfd* deletion, or by chemical inhibitors of the Bfr–Bfd complex, leads to nearly irreversible iron accumulation in Bfr and cytosolic iron limitation, which is often manifested as pyoverdine overproduction.^22,24,26,27^ In this context, it is notable that the FtnA and BfrB subunits are enriched in cells treated with KM-5-35 (Figure 3), likely reflecting the irreversible accumulation of Fe^3+^ in Bfr. The surprising enrichment of Bfr and Ftn subunits in the treated cells suggests that the turnover of iron-loaded Bfr in *P. aeruginosa* may be lower than that of iron-free Bfr, thereby limiting uncontrolled iron release into the cytosol upon degradation, an issue that clearly warrants further investigation.

**Figure 3:**
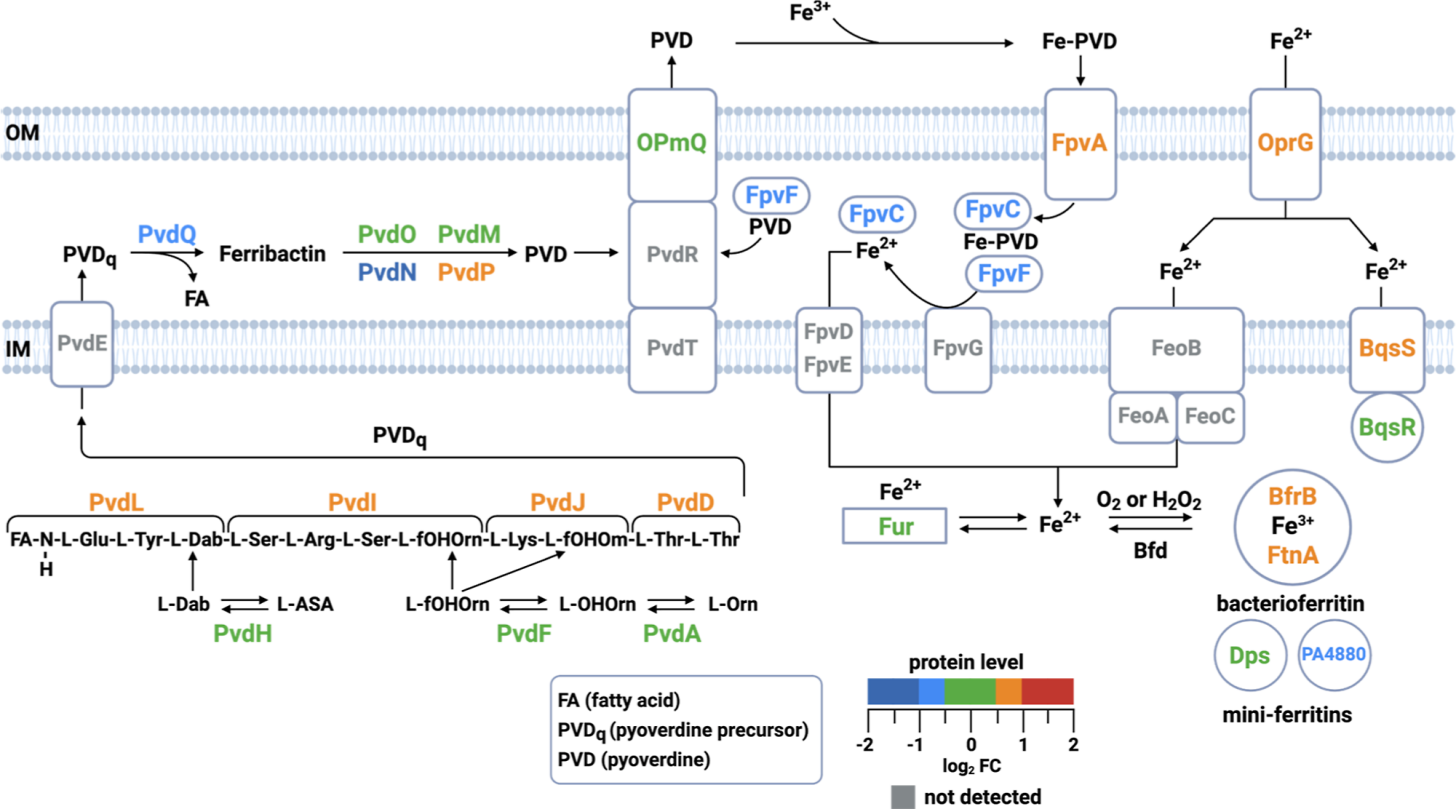
Proteins involved in pyoverdine (PVD) biosynthesis, Fe-PVD utilization, and Fe^2+^ uptake. The schematic shows the assembly and transport of PVD to the extracellular environment, where it binds Fe^3+^ with high affinity. The Fe-PVD complex is then transported across the outer membrane into the periplasmic space, where the coordinated Fe^3+^ is reduced, and the released Fe^2+^ is transported to the cytosol, while PVD is recycled back to the extracellular environment. Fe^2+^ uptake is thought to occur via OprG (outer membrane) and FeoB (inner membrane), with sensing of intracellular Fe^2+^ mediated by the BqsRS two-component system. In the cytosol, a dynamic equilibrium exists between free Fe^2+^, and Fe^3+^ compartmentalized in Bfr, which buffers Fe^2+^ concentrations at levels compatible with Fur-mediated sensing. Enzymes detected as differentially abundant are indicated, and their relative abundances (treated/untreated) are color-coded according to the fold change (log_2_FC) scale.

The irreversible accumulation of iron in Bfr is expected to result in low cytosolic iron levels, triggering iron limitation responses, such as pyoverdine (PVD) overproduction. PVD is a siderophore secreted to sequester environmental Fe^3+^ and facilitate its uptake. PVD biosynthesis and utilization involve two gene clusters: *pvd* (for assembly and export) and *fpv* (for ferripyoverdine uptake and recycling) (Figure 3).^30–32^ In the PVD biosynthesis pathway, the nonribosomal peptide synthetases PvdL, PvdI, PvdJ and PvdD carry out the assembly of the precursor siderophore PVDq, which starts with the attachment of a C_14_ fatty acid to l-Glu and ends after incorporation of the last l-Thr. PVDq is then translocated to the periplasm by PvdE where the myristoleic acid is removed by the acylase PvdQ before maturation of the ferribactin chromophore into PVD by enzymes PvdO, PvdM, PvdN, and PvdP. Mature PVD is then secreted by PvdRT-OpmQ to the extracellular space where it binds Fe^3+^. The resulting ferripyoverdine (Fe-PVD) is recognized and internalized to the periplasm by FpvA, followed by the reduction of Fe^3+^ to Fe^2+^ by proteins FpvCFG and the subsequent transport of Fe^2+^ to the cytosol by FpvDE. The proteomic profile shows enrichment of key proteins (PvdDIJL) required for PVDq assembly, as well as for recognition and import of Fe-PVD in the surviving cells treated with KM-5-35, suggesting iron limitation. However, the enzyme involved in myristoleic acid removal (PvdQ) is depleted, as is PvdN, indicating a potential bottleneck in the process of PVD maturation. Deletion of *pvdQ* abolishes pyoverdine production without affecting growth,^33^ consistent with its essential biosynthetic role. In the context of impaired PVD biosynthesis, it is also noteworthy that several enzymes that function in fatty acid biosynthesis are depleted in the surviving cells treated with KM-5-35 (see Figure 7), which suggests an additional limitation in PVDq biosynthesis. Regarding Fe-PVD utilization and recycling, it is of note that the associated periplasmic enzymes (FpvC, FpvF) are depleted in the treated cells. Collectively, these findings suggest that in the cells surviving KM-5-35 treatment PVD-mediated iron acquisition is impaired by bottlenecks in PVD biosynthesis, maturation, and recycling pathways.

Additional evidence indicating iron limitation is seen in the enrichment of OprG and BqsS (Figure 3). OprG, a porin homologous to *A. baumannii* OmpW, is involved in Fe^2+^ transport,^34^ and upregulated under the microaerophilic to anaerobic conditions encountered in biofilms, where Fe^2+^ is favored.^35^ BqsS, part of the BqsR/BqsS two-component system, senses extracellular Fe^2+^ in *P. aeruginosa*.^36,37^ Other signs of iron starvation include the depletion of iron-dependent enzymes of the TCA cycle aconitase, succinate dehydrogenase, see Figure 5, and Fe-dependent ribonucleotide reductase β-subunit and Fe-dependent superoxide dismutase (SodB) (see Table S1).

### Quorum Sensing

In *P. aeruginosa*, quorum sensing depends on an interconnected regulatory network of three major components, PQS, rhamnolipids, and phenazines. In this context, it is interesting that the surviving biofilm cells displayed a suggestive pattern of phenazine metabolism rewiring, enrichment of Rhl-associated rhamnolipid biosynthesis, and no changes in PQS biosynthesis (Table S3).

Selective remodeling of the phenazine biosynthetic pathway can be gleaned from the information in (Figure 4) and Table S3. Although several proteins involved in phenazine biosynthesis were not detected, the changes observed in some of the detected enzymes (Figure 4) suggest selective remodeling of the phenazine biosynthetic pathway: Enrichment of PhzA2 and PhzC1, together with unchanged levels of PhzD1, PhzE1, and PhzF2, indicates that upstream phenazine precursor synthesis from chorismate remained active. Depletion of PhzB1 and PhzB2 indicates disruption of phenazine-1-carboxylic acid (PCA) assembly within the *phz*1/*phz*2 operons.^38–40^ Concurrent enrichment of PhzM and depletion of PhzS is notable because pyocyanin (PYO) biosynthesis requires sequential activity of the methyltransferase PhzM and the flavin-dependent monooxygenase PhzS, such that depletion of PhzS may limit terminal PYO production despite increased PhzM abundance.^38,40^ Since PYO is a highly redox-active molecule involved in oxidative stress generation and extracellular electron transfer, reduced PYO synthesis may represent an adaptive response to KM-5-35-induced stress.^41^ In this context, the concomitant enrichment of PhzH, which diverts PCA toward phenazine-1-carboxamide (PCN), suggests redistribution of phenazine flux away from PYO and toward alternative phenazines. This redistribution toward PCA, and PCN may be useful to overcome the KM-5-35-induced iron limitation because PCA and PCN can reduce Fe^3+^ to Fe^2+^ efficiently, compared to the relatively poor Fe^3+^ reducing ability of PYO.^42^

**Figure 4:**
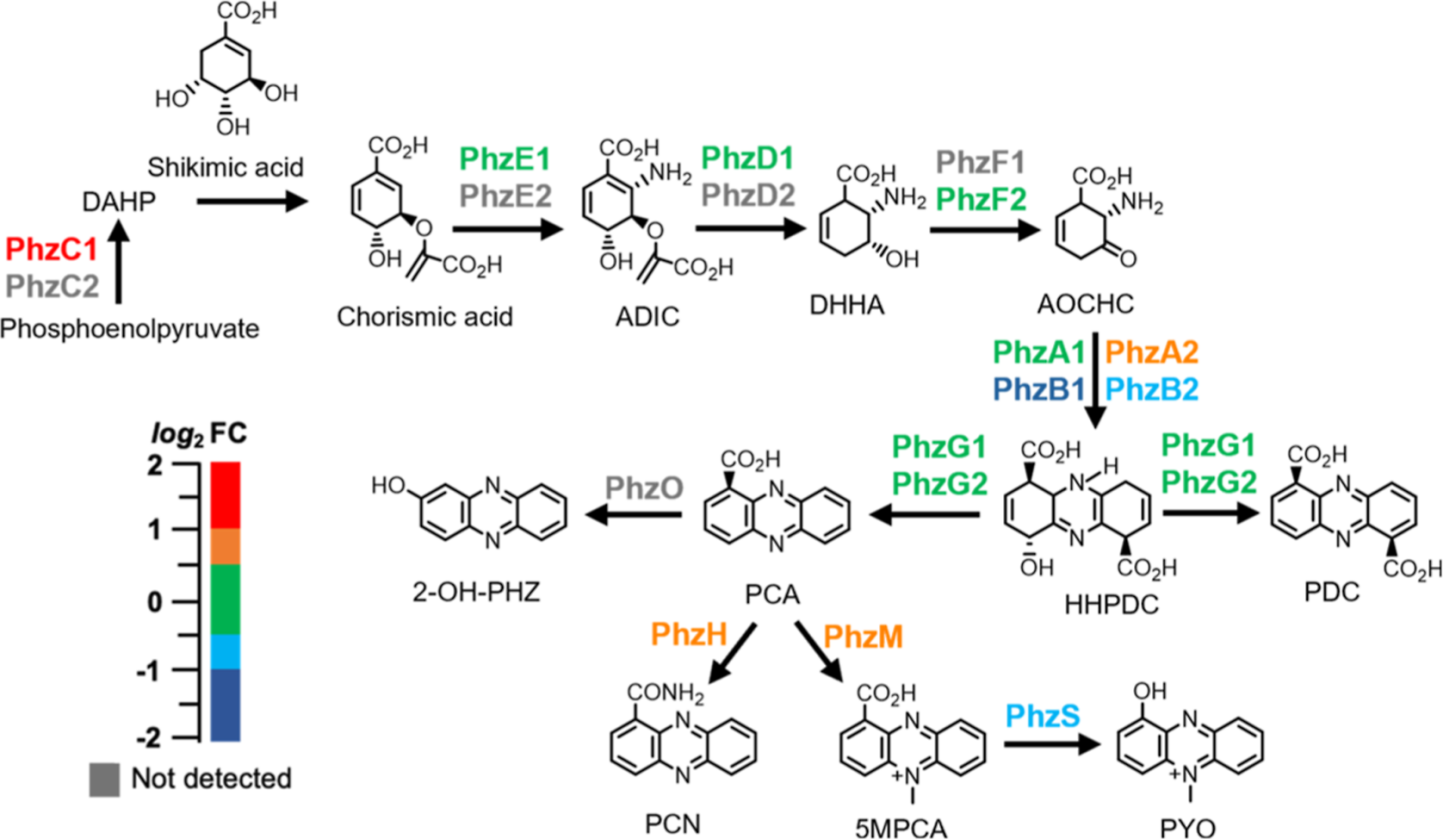
Schematic representation of phenazine biosynthesis in *P. aeruginosa*. The schematic illustrates the biosynthesis of phenazines carried out by enzymes encoded by the two operons, *phz1* and *phz2*. Enzymes detected as differentially abundant are indicated, and their relative abundances (treated/untreated) are color-coded according to the fold change (log_2_FC) scale.

RhlI, RhlA, and RhlB are enriched (Table S3), indicating enhanced production of biosurfactants in the surviving cells treated with KM-5-35. RhlI is an autoinducer synthase that produces the quorum sensing signal molecule C4-HSL (*N*-butyryl-l-homoserine lactone), which binds to the transcriptional regulator RhlR, activating expression of genes involved in biofilm development and rhamnolipid production. RhlA catalyzes the synthesis of β-hydroxy fatty acid precursor (HAA), while RhlB transfers a rhamnose sugar onto the HAA lipid moiety to form monorhamnolipids. Given that these enzymes are regulated by quorum sensing and environmental stress responses and are central to biofilm architecture and structural adaptation,^43–45^ their enrichment in the surviving cells suggests enhanced production of biosurfactants that may function in biofilm remodeling and stress response.

Interestingly, the proteomic profile of the surviving cells shows no changes in PQS biosynthesis proteins (Table S3), which indicates that upstream quinolone quorum sensing signals remain stable, and implies that the shifts in phenazine and rhamnolipid pathways arise from downstream metabolic reprogramming rather than altered PQS signal production.^46,47^

Collectively, the observations support a model in which surviving cells treated with KM-5-35 undergo selective metabolic rerouting of quorum sensing, with stable PQS signaling, but rerouting of phenazine metabolism toward PCN- and 5MPCA-associated branches and concurrent enhancement of rhamnolipid-mediated biofilm restructuring. These metabolic shifts may facilitate Fe^2+^ uptake, stress response, and survival.

### Carbon Metabolism and Amino Acid Biosynthesis are Affected by KM-5-35

Comparison of the proteomic profiles from KM-5-35-treated cells and untreated biofilm cells revealed significant alterations in central carbon metabolism, including the TCA cycle and the synthesis of several amino acids (Figure 5). Although *Pseudomonas* catabolize glucose via the Enter-Doudoroff (ED), Embden-Meyerhof-Parnas (EMP), and the pentose phosphate (PP) pathways, the lack of a 6-phosphofructo-1-kinase, which catalyzes the conversion of fructose-6-P (F6P) to fructose-1,6 P (FBP), precludes the typical EMP pathway, making ED the dominant pathway for hexose degradation.^48^ The ED pathway merges with the upper EMP path for recycling glyceraldehyde-3P (G3P) to fuel biomass production and alginate; recycling of G3P occurs via the stepwise sequence G3P → Glycerone-P (DHAP) → FBP → F6P → Glucose-6P (G6P)^49^ (Figure 5).

**Figure 5:**
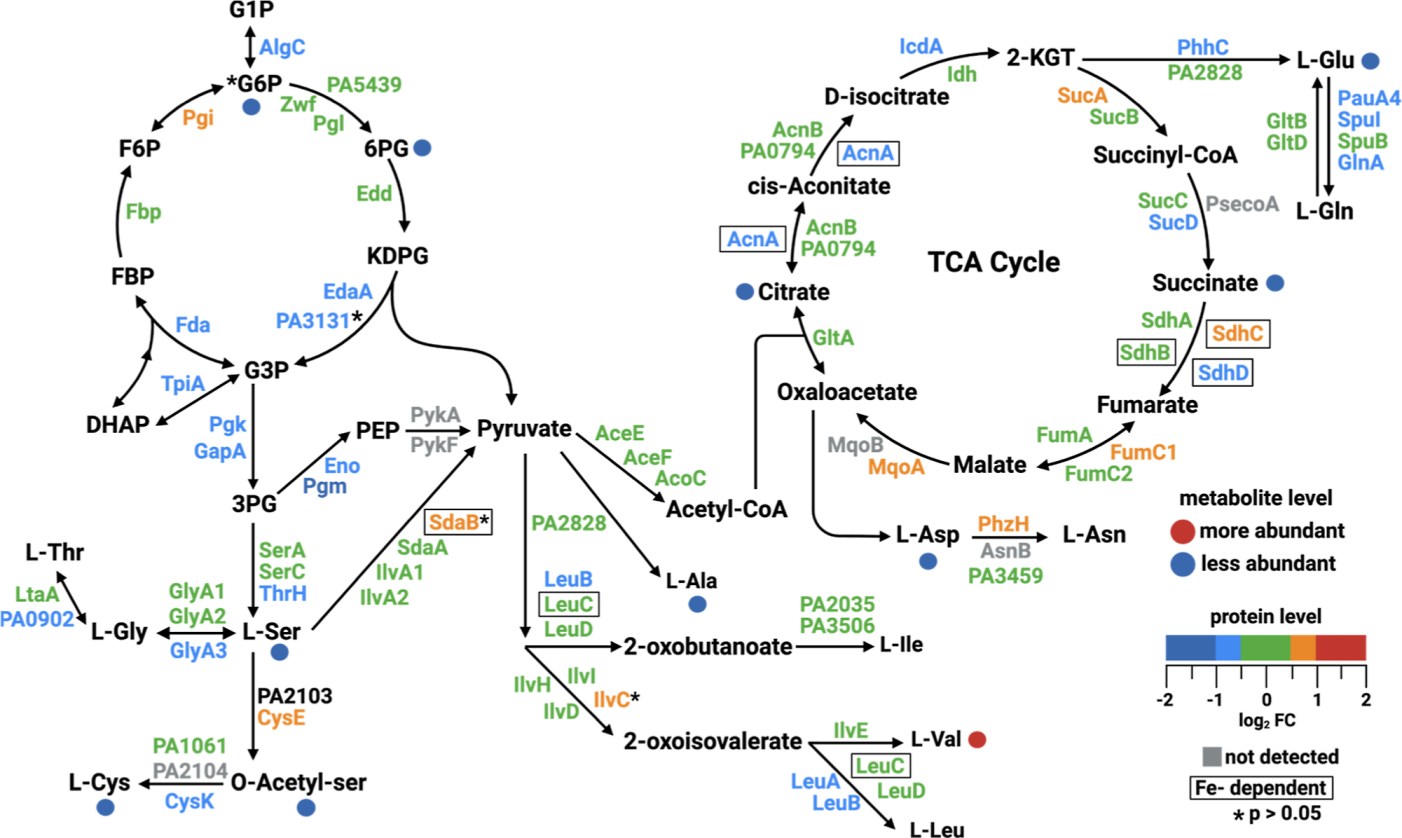
Schematic representation of glucose catabolism and tricarboxylic acid cycle in *P. aeruginosa*. The schematic illustrates the main carbon metabolic pathways and selected amino acid biosynthesis routes in *P. aeruginosa*, annotated with enzymes identified as affected in the proteomic profiling of biofilm cells treated with KM-5-35. Enzymes detected as differentially abundant are indicated, and their relative abundances (treated/untreated) are color-coded according to the fold change (log_2_FC) scale. Only metabolites authentically detected by untargeted metabolomics analysis are highlighted by circles adjacent to the metabolite name; the circles are color coded to reflect relative abundance in the treated cells compared to untreated controls.

In KM-5-35-treated cells, enzymes of the ED pathway responsible for converting KDPG to pyruvate and G3P (Eda, PA3131) are depleted, as are enzymes involved in subsequent steps: conversion to 3PG (GapA, Pgk), to PEP (Pgm, Eno), and recycling G3P to FBP (TpiA, Fda). The depletion of these enzymes suggests bottlenecks in pyruvate synthesis, which connects glycolysis to the TCA cycle. Furthermore, the reduced abundance of these enzymes extends to the biosynthesis of amino acids derived from glycolytic and TCA cycle intermediates, such as serine and cysteine (Figure 5), likely due to limited 3PG availability and associated enzymatic deficiencies.

Pyruvate, the end product of glycolysis, is converted into acetyl-CoA and enters the TCA cycle by condensing with oxaloacetate to produce citrate. In treated cells, the levels of citrate and of the enzymes for the sequential conversion of citrate to *cis*-aconitate, isocitrate, α-ketoglutarate, succinate, and fumarate (AcnA, IcdA, SucD, and SdhD, respectively), are depleted. These deficiencies probably give rise to the depleted status of amino acids synthesized from TCA cycle metabolites, such as Asp (from oxaloacetate), and Glu (from α-ketoglutarate, 2-KTG). Similarly, alanine, which is derived mainly from serine via pyruvate, is depleted in the KM-5-35-treated cells. In aggregate, KM-5-35 treatment affects core metabolic pathways, probably affecting both energy production and biosynthesis of amino acids.

### Cofactor Biosynthesis is Affected by Treatment with KM-5-35

#### Heme Biosynthesis

Heme is an essential cofactor of several critical enzymes, including heme dependent catalases, and various heme dependent enzymes involved in electron transfer reactions that support respiration and other important metabolic processes.^50,51^ *P. aeruginosa* can obtain heme from its host or synthesize it de novo via the C5 pathway.^50^ In this pathway glutamine tRNA synthetase (GltX) catalyzes the formation of glutamyl *t*-RNA, which is subsequently converted to glutamate-1-semialdehyde (GSA) by HemA (Figure 6A). GSA is then transformed into 5-aminolevulinic acid (ALA), the first committed precursor in the heme biosynthetic pathway, by HemL. Through a series of enzymatic reactions ALA is converted to protoporphyrin IX, after which ferrochelatase (HemH) inserts Fe^2+^ into protoporphyrin IX to produce protoheme IX (heme). Notably, in cells treated with KM-5-35, the levels of glutamate, as well as those of HemL, HemC and HemF, are depleted (Figure 6A), while HemH is enriched. The reduction of glutamate and multiple biosynthetic enzymes is expected to limit protoporphyrin IX production, and the enrichment of HemH suggests that iron limitation leads to an accumulation protoporphyrin IX, further impairing heme biosynthesis.

**Figure 6:**
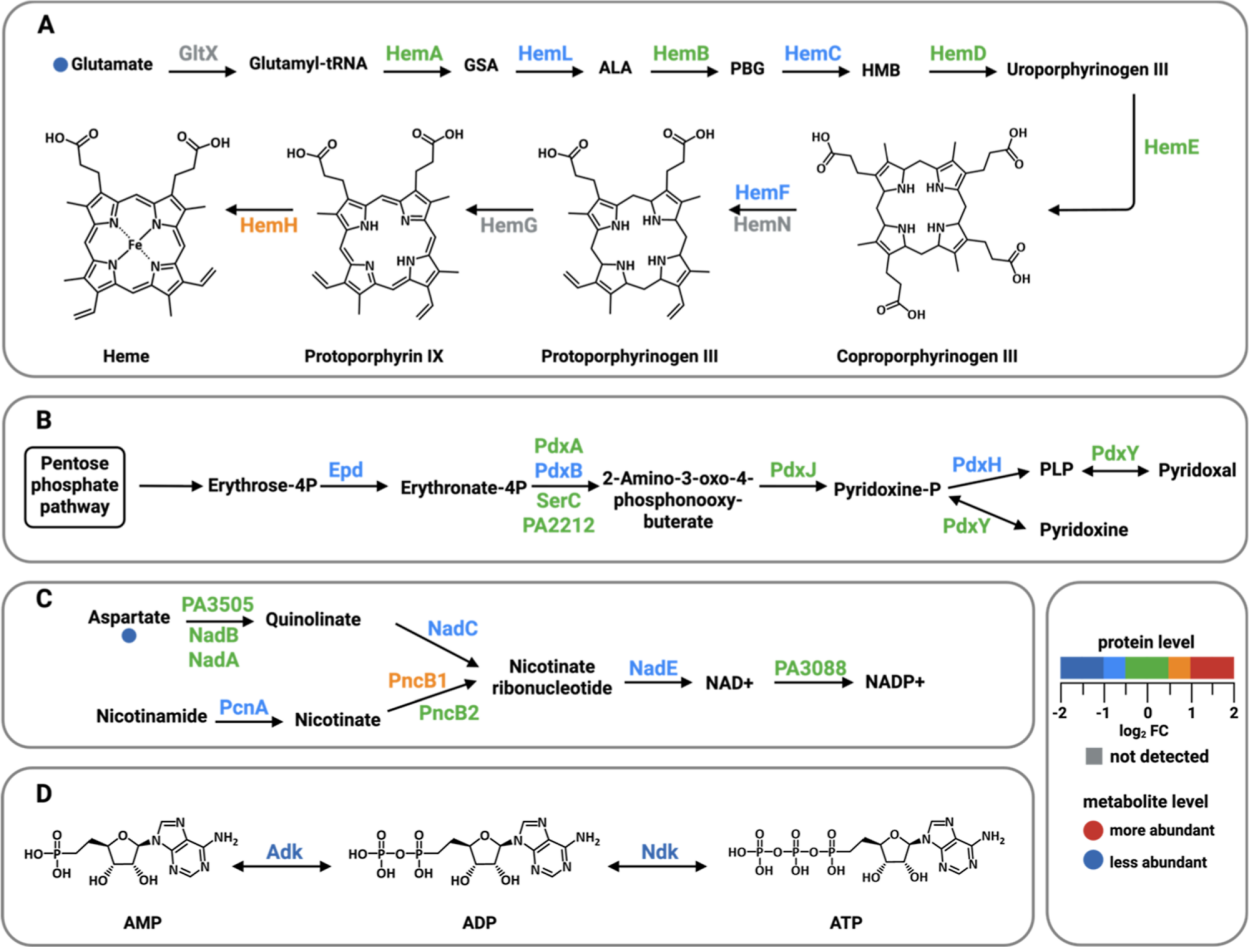
Schematic representation of essential cofactor biosynthesis in *P. aeruginosa*. The schematic illustrates the biosynthetic pathways for several essential cofactors: (A) heme, (B) pyridoxal 5-phosphate (PLP), (C) NAD/NADP, including de novo synthesis from aspartate and the salvage pathway from nicotinamide, (D) AMP, ADP, and ATP. The relative abundances (treated/untreated) of proteins included in the scheme are color coded according to the fold change (log_2_FC) scale. Only metabolites authentically detected by untargeted metabolomics analysis are highlighted by circles adjacent to the metabolite name; the circles are color coded to reflect relative abundance in the treated cells compared to untreated controls.

#### Pyridoxal-5′-Phosphate (PLP) Biosynthesis

PLP-dependent enzymes catalyze a wide-range of reactions, including transamination, decarboxylation, racemization, elimination and replacement of electrophilic groups in the biosynthesis of amino acids and amino sugars.^52^ Gamma proteobacteria, including *P. aeruginosa*, synthesize PLP via the PdxA/PdxJ pathway (Figure 6B), involving the enzymes PdxA, PdxB, PdxJ, and PdxH. Several of these enzymes are considered promising targets for the development of new antibacterial agents.^53^ In KM-5-35-treated cells, the NADH-dependent enzyme PdxB, as well as PdxH, are depleted, which may lead to reduced intracellular PLP levels.

#### NAD^+^, NADP^+^, and ATP Biosynthesis

In *P. aeruginosa* NAD^+^ is synthesized de novo from aspartate and via salvage pathways that utilize nicotinamide or nicotinate scavenged from the environment^54,55^ (Figure 6C). In the KM-5-35-treated cells, levels of the aspartate precursor, as well as NadC and NadE, which convert quinolinate to nicotinate ribonucleotide and then to NAD^+^, are decreased. In the salvage pathway, the enzymes PcnA and NadE are also depleted. Together, these findings suggest that treatment with KM-5-35 may reduce cellular NAD^+^/NADH pools, compromising the numerous metabolic transformations carried out by NAD­(P)­H-dependent enzymes. Similarly, enzymes Adk and NdK, which are required for the biosynthesis of AMP, ADP and ATP are depleted in cells treated with KM-5-35 (Figure 6D).

### Fatty Acid Biosynthesis is Affected by Treatment with KM-5-35

Fatty acid biosynthesis generates the phospholipids and lipoproteins necessary for cell membrane integrity, as well as important cofactors such as lipoate and biotin, and diffusible metabolites such as quorum sensing molecules and siderophores (e.g., PVD).

In most bacteria, including *P. aeruginosa*, de novo fatty acid biosynthesis proceeds via the type II fatty acid system (FASII), where the nascent fatty acid chain is assembled on a freely diffusible phosphopantetheinylated acyl carrier protein (ACP).^56,57^ The FASII pathway also provides intermediates such as the β-hydroxyl myristoyl-ACP and lauryl-ACP needed for lipopolysaccharide biosynthesis.^58^ Fatty acids are assembled by the sequential addition of two-carbon units through the condensation of a malonyl-ACP and a growing acyl acceptor (Figure 7). The pathway begins with the acetyl-CoA carboxylase complex (AccABCD), which converts acetyl-CoA and bicarbonate into malonyl-CoA, which is then transferred to the ACP by FabD to generate malonyl-ACP. In *P. aeruginosa*, FabY catalyzes the condensation of malonyl-ACP with acetyl-CoA to produce β-ketoacyl-ACP,^57^ which enters the elongation cycle. During elongation, the β-keto group is reduced to a hydroxyl group by the NADPH-dependent enzyme FabG. The resulting β-hydroxyacyl-ACP is dehydrated to *trans*-2-enoylacyl-ACP by FabZ or FabA,^59^ and then reduced to saturated acyl-ACP by the NADPH-dependent enoyl reductase FabI,^60^ or the triclosan-resistant isoenzyme FabV.^61^ The elongated acyl-ACP intermediate is condensed with another malonyl-ACP by the elongation synthases FaB or FabF, generating a new β-ketoacyl-ACP that reenters the reduction cycle.^62^ Note that several of the enzymes involved in the initiation and elongation cycle (FabD, FabI, FabV, and FabB) are depleted in the KM-5-35-treated cells (Figure 7). This depletion suggests the presence of metabolic bottlenecks that likely impair acyl chain biosynthesis required for membrane phospholipids and lipopolysaccharide production.

**Figure 7:**
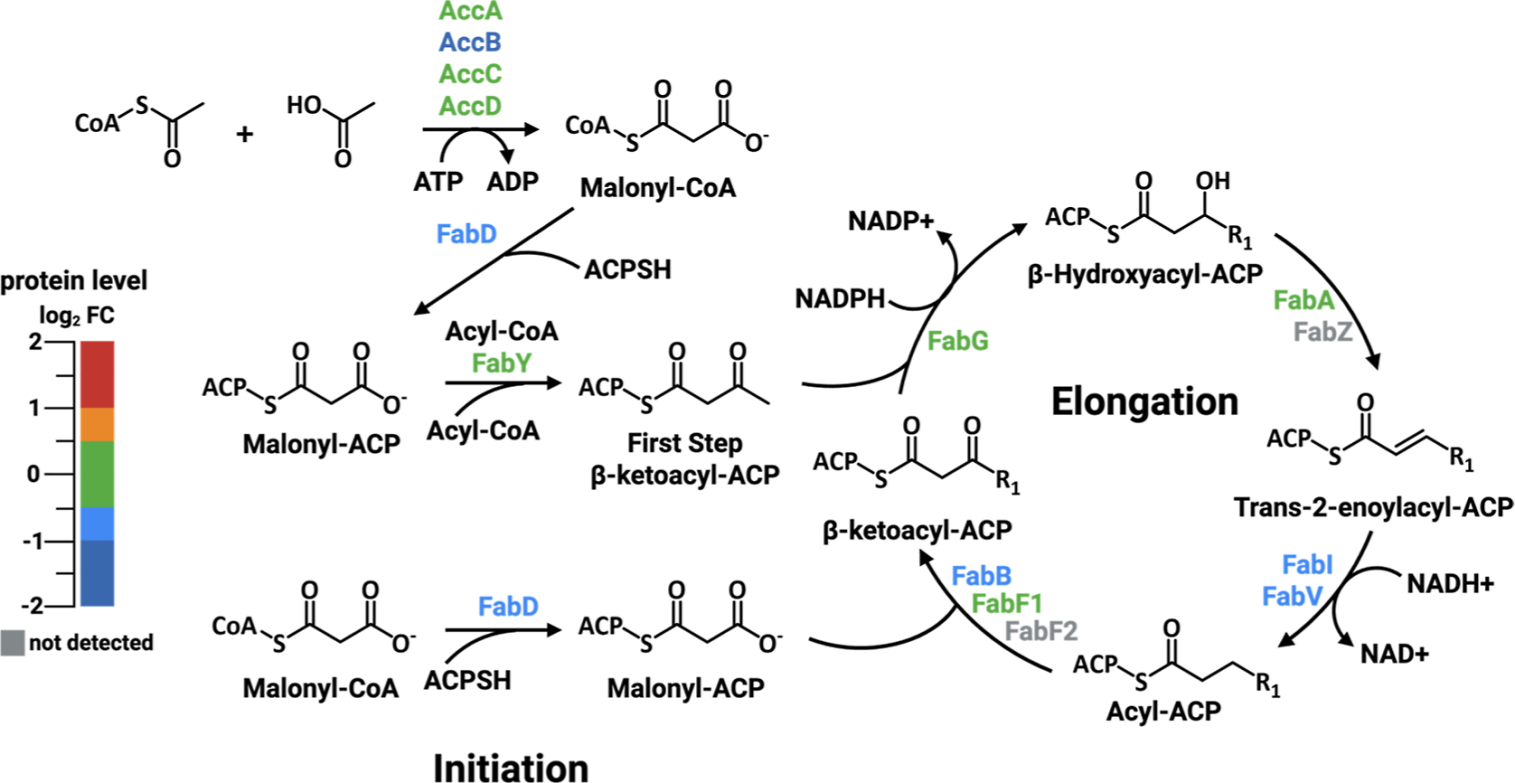
Schematic depicts the type II fatty acid synthesis system, which consists of an initiation step followed by repeated elongation steps catalyzed by the Fab family of enzymes. Several Fab enzymes are depleted in cells surviving KM-5-35 treatment. The relative abundances of these proteins (treated/untreated) are color coded according to the fold change (log_2_FC) scale. No metabolites were detected.

### Impact of KM-5-35 on Essential Proteins and Cellular Processes

A screen conducted across six different media identified 352 general essential genes in *P. aeruginosa* encompassing fundamental cellular processes such as DNA replication, transcription, translation, protein folding, and the biosynthesis of cofactors, lipids and peptidoglycan.^63^ Of these, 83 essential proteins were significantly affected in KM-5-35-treated cells compared to untreated controls (Table 1). The impacted proteins are involved in DNA replication, transcription and translation, lipid and lipopolysaccharide biosynthesis, membrane biogenesis, cofactor biosynthesis, as well as energy and metabolism.

**Table 1:** Essential Proteins Showing Statistically Significant Differential Abundance in KM-5-35-Treated *P. aeruginosa* Cells Relative to Untreated Controls

Locus ID	name	product	log_2_ FC
**DNA replication, translation, and repair**			
PA0001	*dnaA*	chromosomal replication initiator protein DnaA	0.62
PA0002	*dnaN*	DNA polymerase III, beta chain	–0.74
PA0019	*def*	polypeptide deformylase	–0.85
PA0577	*dnaG*	DNA primase	0.54
PA0579	*rpsU*	30S ribosomal protein S21	–1.27
PA1794	*gln S*	glutaminyl-*t*RNA synthetase	–0.71
PA2612	*serS*	seryl-*t*RNA synthetase	–0.55
PA2619	*infA*	initiation factor	–0.68
PA2743	*infC*	translation initiation factor IF-3	–0.54
PA2961	*holB*	DNA polymerase III, delta prime subunit	0.55
PA3162	*rpsA*	30S ribosomal protein S1	–0.76
PA3653	*frr*	ribosome recycling factor	–1.3
PA3655	*tsf*	elongation factor Ts	–1.17
PA3987	*leuS*	leucyl-*t*RNA synthetase	–0.53
PA4252	*rplX*	50S ribosomal protein L24	–1.15
PA4257	*rpsC*	30S ribosomal protein S3	0.61
PA4266	*fusA1*	elongation factor G	–0.8
PA4268	*rpsL*	30S ribosomal protein S12	0.55
PA4269	*rpoC*	DNA-directed RNA polymerase beta* chain	0.56
PA4484	*gatB*	Glu-tRNA(Gln) amidotransferase subunit B	–0.67
PA4563	*rpsT*	30S ribosomal protein S20	–0.86
PA4744	*infB*	translation initiation factor IF-2	–0.52
PA4931	*dnaB*	replicative DNA helicase	0.82
PA5316	*rpmB*	50S ribosomal protein L28	0.57
PA5492	-	conserved hypothetical protein	0.58
**Adaptation, protection, chaperones & Heat shock proteins**			
PA0594	*surA*	peptidyl-prolyl cis–trans isomerase SurA	–0.99
PA1151	*imm2*	pyocin S2 immunity protein	–0.96
PA3810	*hscA*	heat shock protein HscA	–0.68
PA4366	*sodB*	superoxide dismutase	–1.35
PA4385	*groEL*	groEL protein	0.87
PA4566	*obg*	GTP-binding protein Obg	–0.69
**Fatty acid, LPS, and capsule biosynthesis**			
PA0341	*lgt*	prolipoprotein diacylglyceryl transferase	0.99
PA0971	*tolA*	TolA protein	–1.67
PA0972	*tolB*	TolB protein	–0.81
PA1162	*dapE*	succinyl-diaminopimelate desuccinylase	–0.83
PA1165	*pcpS*	PcpS	0.71
PA2614	*lolA*	periplasmic chaperone LolA	–1.18
PA2968	*fabD*	malonyl-CoA-[acyl-carrier-protein] transacylase	–0.78
PA3117	*asd*	aspartate semialdehyde dehydrogenase	–0.70
PA3636	*kdsA*	2-dehydro-3-deoxyphosphooctonate aldolase	0.58
PA3984	*lnt*	apolipoprotein N-acyltransferase	0.61
PA4406	*lpxC*	UDP-3-O-acyl-N-acetylglucosamine deacetylase	–0.99
PA4411	*murC*	UDP-N-acetylmuramate--alanine ligase	0.51
PA4425	*gmhA*	sedoheptulose 7-phosphate isomerase GmhA	–0.61
PA4749	*glmM*	phosphoglucosamine mutase	0.54
PA4847	*accB*	biotin carboxyl carrier protein (BCCP)	–1.57
PA4998	-	conserved hypothetical protein	0.58
**Biosynthesis of cofactors**			
PA0342	*thyA*	thymidylate synthase	–0.68
PA0546	*metK*	methionine adenosyltransferase	–0.76
PA0956	*proS*	prolyl-*t*RNA synthetase	–0.53
PA1796	*folD*	5,10-methylene-tetrahydrofolate dehydrogenase/cyclohydrolase	–0.80
PA3977	*hemL*	glutamate-1-semialdehyde 2,1-aminomutase	–0.73
PA4051	*thiL*	thiamine monophosphate kinase	0.58
PA4280	*birA*	biotin-protein ligase	0.74
PA4569	*ispB*	octaprenyl-diphosphate synthase	–0.85
PA4655	*hemH*	ferrochelatase	0.71
PA4669	*ipk*	isopentenyl monophosphate kinase	0.72
PA4920	*nadE*	NH3-dependent NAD synthetase	–0.89
PA4728	*folK*	2-amino-4-hydroxy-6-hydroxymethyldihydropteridine pyrophosphokinase	0.94
PA5065	*ubiB*	ubiquinone biosynthetic protein UbiB	0.57
**Energy and metabolism**			
PA1155	*nrdB*	NrdB, tyrosyl radical-harboring component of class Ia ribonucleotide reductase	–1.11
PA3770	*guaB*	inosine-5′-monophosphate dehydrogenase	0.94
PA4031	*ppa*	inorganic pyrophosphatase	–0.94
PA4748	*tpiA*	triosephosphate isomerase	–0.67
PA1585	*sucA*	2-oxoglutarate dehydrogenase (E1 subunit)	0.74
PA2951	*etfA*	electron transfer flavoprotein α-subunit	–1.25
PA2952	*etfB*	Electron transfer flavoprotein β-subunit	–1.17
PA2953	-	electron transfer flavoprotein-ubiquinone oxidoreductase	0.58
PA5558	*atpF*	ATP synthase B chain	0.62

#### Disruption of Membrane Biosynthesis and Envelope Integrity

KM-5-35 treatment notably affects several essential proteins involved in membrane biosynthesis and envelope integrity. For example, TolA, TolB and OprL, components of the TolA-Pal system (TolA, TolB, TolQ, TolR, and Pal/OprL), are critical for maintaining envelope integrity by spanning the periplasm and connecting the inner and outer membranes. Depletion of TolB in *P. aeruginosa* results in compromised cell-envelope integrity and increased susceptibility to several antibiotics.^64^ Similarly, SurA, a periplasmic chaperone essential for folding and shuttling outer membrane proteins (OM) across the periplasm, is depleted. Depletion of SurA in *P. aeruginosa* affects OM integrity and increases antibiotic susceptibility.^65^ LolA, which transports lipoproteins across the periplasm to the OM receptor LolB, is also affected; its function is essential for OM integrity and is considered a target for developing antibacterial inhibitors.^66^ Similarly, LpxC, which catalyzes the first committed step in lipid A biosynthesis and represents a target for new antibacterial development,^67^ is depleted.

#### Effects on Ribosomal Proteins and Translational Activity

Proteomic analysis of the cells surviving treatment with KM-5-35 also reveals significant changes in proteins associated with both the small (30S) and large (50S) ribosomal subunits (Table 1). Notably, the ribosome recycling factor Frr (required for the release of ribosomes from mRNA at the end of protein synthesis), the translation initiation factor 3 (InfC, which binds to the 30S ribosomal subunit and is essential for protein synthesis), the elongation factor G (FusA, required for translocation during protein synthesis), and the elongation factor Ts (Eft), are all depleted in the cells surviving treatment with KM-5-35. This pattern suggests that surviving cells are in a state of reduced translation activity, likely reflecting a state of translational stress.

### Synergistic Killing of Biofilm Cells by KM-5-35 and Aminoglycosides

The proteomic profile of cells surviving treatment with KM-5-35 indicates significant intracellular stress, including central carbon metabolism, amino acid biosynthesis, and fatty acid biosynthesis, as well as disruption in ribosome homeostasis, with depletion of Frr and several translation initiation and elongation factors (InfA, InfB, InfC, FusA, Tsf) (Table 1). This metabolic downshift may lead to an erratic or unstable proton motive force (PMF) and impaired ribosome turnover and decreased translational flux, which may be exacerbated by aminoglycosides. To investigate this, we evaluated the effects of KM-5-35 in combination with three aminoglycosides (gentamicin, amikacin, and tobramycin) and with a β-lactam antibiotic (Meropenem). As shown in Figure 8, *P. aeruginosa* biofilms are susceptible to KM-5-35 (50 μM), but display tolerance to gentamicin, amikacin, tobramycin and Meropenem at 20× MIC. Remarkably, the combination of KM-5-35 and gentamicin, or KM-5-35 and amikacin, is highly synergic resulting in only 0.04%, or 0.08% survival, respectively. In this context, it is important to underscore that the differential potentiation observed with gentamicin and amikacin relative to tobramycin and Meropenem argues against nonspecific envelope disruption caused by KM-5-35.

**Figure 8:**
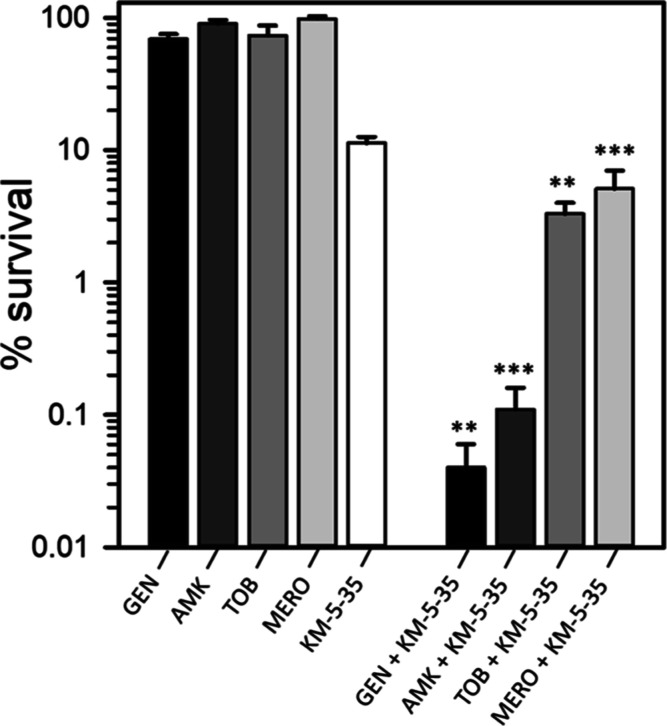
Mature biofilms were treated for 24 h with either 20× MIC gentamicin (GEN), 20× MIC amikacin (AMK), 20× MIC tobramycin (TOB), 20× MIC Meropenem (MERO), or 50 μM (14 μg/mL) KM-5-35. Combination treatments consisted of 50 μM KM-5-35 with 20× MIC GEN, AMK, TOB, or MERO. Percent survival was calculated as the ratio of CFU/mL after treatment to CFU/mL before treatment. MIC values were as follows: GEN, 16 μg/mL; AMK, 45 μg/mL; TOB, 8 μg/mL; MERO, 0.5 μg/mL. Data represent the mean percent survival from three biological replicates, each performed in triplicate. Statistical significance for combination treatments compared to antibiotic alone is indicated by *** (*p* < 0.001) or ** (*p* < 0.01).

Aminoglycosides are known to induce translational errors and impede ribosome recycling by stabilizing intersubunit interactions that oppose Frr-dependent ribosomal splitting.^68^ Aminoglycoside binding of ribosomes can also induce single and cluster errors (up to four amino acid substitutions) in protein sequences, leading to the synthesis of aggregation-prone proteins, increased proteotoxicity, and autocatalytic drug uptake that culminates in cell death. The efficacy of distinct aminoglycosides in inducing proteotoxic error clusters depends on the drug-induced ribosomal conformation and the residence time of the aminoglycoside on the ribosome relative to the translocation rate.^69^ Hence, it is possible that under the stress conditions induced by KM-5-35, gentamicin and amikacin may more effectively exploit deficiencies in ribosome recycling and impaired efflux compared to tobramycin. In *P. aeruginosa*, the MexXY-OprM is a major efflux system for aminoglycosides.^70^ While its expression can be induced by ribosomal stress, its activity depends on the PMF.^71^ KM-5-35-treated cells show enrichment of MexX, but no change in OprM (Table S4), along with evidence of compromised PMF, as indicated by depletion of proteins involved in central carbon metabolism, TCA cycle-linked amino acid biosynthesis (Figure 5), fatty acid biosynthesis (Figure 7), and NAD and ATP biosynthesis (Figure 6). These findings, which indicate a metabolic downshift, likely limit PMF and, consequently, the functional capacity of efflux pumps.

It is noteworthy that tobramycin, the aminoglycoside with the greatest activity against planktonic *P. aeruginosa* based on MIC values, exhibited the least synergy with KM-5-35 against biofilms. Although the mechanisms underlying the differential synergy observed among the aminoglycosides are likely multifactorial, it is possible that the physiological state induced by KM-5-35 only modestly enhances biofilm susceptibility to tobramycin, while substantially increasing the access and/or efficacy of gentamicin and amikacin. Consequently, the well-established tolerance of *P. aeruginosa* biofilms to aminoglycosides can be overcome in vitro through combination treatment with KM-5-35. Whether this combination will prove effective for eradicating biofilm infections in vivo remains unknown; however, the promising results presented here suggest that the recalcitrance of biofilms to aminoglycosides may be mitigated by compounds such as KM-5-35, which can disrupt biofilm-associated defenses in *P. aeruginosa* and create physiological conditions that permit certain aminoglycosides to exert their bactericidal activity.

## Conclusions

The development of new compounds targeting previously unexploited bacterial pathways holds significant promise for innovative antimicrobial therapies. One such strategy involves inhibiting the mobilization of iron from Bfr, capitalizing on the essentiality of iron, especially under the iron-limited conditions imposed by host immune response. Iron mobilization from Bfr requires the binding of its cognate ferredoxin, Bfd. Disruption of the Bfr–Bfd complex, as seen in *P. aeruginosa* strains lacking the *bfd* gene, leads to irreversible accumulation of unusable iron within Bfr, cytosolic iron depletion,^22^ metabolic dysregulation in planktonic cells,^23^ and impaired biofilm maturation.^24^ A search for small molecule inhibitors of the Bfr–Bfd complex identified 4-aminoisoindoline-1,3-dione derivatives, such as KM-5-35. These compounds can penetrate *P. aeruginosa* and *A. baumannii* cells, bind Bfr, and inhibit iron mobilization.^27^ Notably, while these derivatives are bacteriostatic against planktonic *P. aeruginosa*, they exhibit bactericidal activity against cells within biofilms.^26,27^

This study focused on elucidating the effects of KM-5-35 cells on mature *P. aeruginosa* biofilms. Treatment of mature biofilms with 50 μM KM-5-35 for 24 h resulted in approximately 90% cell death. Proteomic profiling of the surviving cells revealed pronounced metabolic changes, including cytosolic iron depletion, upregulation of enzymes involved in iron uptake, and depletion of iron-dependent enzymes such as NrdB Fe-dependent ribonucleotide reductase β-subunit, SodB (Fe-dependent superoxide dismutase), Fe-dependent TCA enzymes like aconitase (AcnA) and succinate dehydrogenase (SdhA). The iron limitation induced by KM-5-35 in surviving cells was accompanied by cytosolic stress, including impaired ribosome translocation and recycling, disrupted central carbon metabolism, and reduced fatty acid and amino acid biosynthesis. These metabolic disturbances probably result in an unstable PMF and diminished efflux capacity. In this energetically compromised state, the aminoglycosides gentamicin and amikacin can efficiently exploit the translational stress induced by KM-5-35, leading to profound synergistic killing of biofilm cells.

## Experimental Section

Unless otherwise specified, all chemicals were purchased from Fisher Scientific. *P. aeruginosa* PAO1 was obtained from the University of Washington Genome Center. *P. aeruginosa* was routinely cultured in Pseudomonas Isolation (PI) medium, composed of 20 g L^–1^ peptone, 0.3 g L^–1^ MgCl_2_·6H_2_O, 10 g L^–1^ K_2_SO_4_, 25 mg L^–1^ triclosan, and 20 mL L^–1^ glycerol, adjusted to pH 7.0. Starter cultures were prepared from a single colony and incubated in 5 mL PI medium supplemented with 10 μM Fe at 37 °C, shaking at 220 rpm for 14 h. Biofilms were exposed to antimicrobial compounds in AB minimal media: 2 g/L (NH_4_)_2_SO_4_, 6 g/L Na_2_HPO_4_, 3 g/L KH_2_PO_4_, 3 g/L NaCl, 0.01 g/L Na_2_SO_4_, 0.4 g/L MgCl_2_, 0.01 g/L CaCl_2_. The media was prepared and autoclaved as previously reported^72^ and prior to use was supplemented with filtered sterilized glucose (3 mM final concentration), (NH_4_)_2_Fe­(SO_4_)_2_ (20 μM final concentration), and trace metals 3 μM CuSO_4_, 7.6 μM ZnSO_4_, 2 μM Co­(NO_3_)_2_, 0.15 μM (NH_4_)_2_MoO_4_, 9.4 μM Na_2_B_4_O_7_ (all are final concentrations from individual autoclaved stocks).

### Biofilm Growth, Treatment with KM-5-35, Combination Treatment, and Sample Preparation for Proteomic Profiling

*P. aeruginosa* PAO1 were grown in PI media supplemented with 20 μM Fe as described previously.^27^ Starter cultures were diluted to OD_600_ = 0.001 in 4 mL of PI media supplemented with 20 μM Fe, transferred to 35 × 10 mm Petri dishes, and incubated statically at 30 °C for 27 h. Pellicle biofilms were transferred gently onto circular (1.5 cm diameter) glass coverslips by contacting the pellicle surface with a coverslip. The coverslip-adhered biofilms were washed three times by floating them on PBS with the biofilm exposed to the liquid. The coverslip-adhered biofilms were then floated on 1.5 mL AB challenge media contained in one well of a 12-well microplate, with the biofilm exposed to the solution for 24 h at 35 °C. AB challenge media is AB minimal media supplemented with 0.025% HPMC, 1.5% DMSO and antimicrobial agent: 50 μM KM-5-35, or antibiotic present at 20× MIC, or the combination of 50 μM and 20× MIC antibiotic. Following treatment, planktonic and loosely attached cells were removed by washing the coverslips three times in 3 mL PBS. To disrupt the extracellular matrix and recover biofilm cells, coverslips were placed in 50 mL conical tubes containing 2 mL of zirconia beads (0.1 mm diameter, BioSpec Products), 10 mL PBS, 0.2 μg/mL alginate lyase, and 0.2 μg/mL DNase, then incubated at room temperature for 3 min, and vortexed vigorously for 4 min. After allowing bead sedimentation, a 100 μL aliquot was taken for serial dilution and colony enumeration of viable cells (CFU/mL) in PIA plates. The remaining cell suspension was pelleted and stored at −80 °C for further analysis.

For proteomic analysis, cell pellets from three biological replicates (three KM-5-35 treated, three DMSO controls) were resuspended in 500 μL of 100 mM triethylammonium bicarbonate buffer (TEAB, pH 8.5) with 2% SDS, and lysed by sonication (Qsonica Q500, 20% pulse amplitude, 20 s total: 5 s pulse-on/55 s pulse-off cycles). Lysate solutions were clarified and stored at −80 °C. Protein concentrations were determined using Pierce BCA Protein Assay Kit (Thermo Scientific) with an eight-point BSA standard curve. Aliquots containing 60 μg protein were used for trypsin digestion: proteins were reduced with 50 mM dithiothreitol (DTT) at 55 °C for 1 h, alkylated with 50 mM iodoacetamide (IAA) at room temperature in the dark for 30 min, and precipitated with 600 μL acetone at −20 °C for 4 h. After centrifugation (8000*g*, 10 min, 4 °C), pellets were air-dried (3 min) and resuspended in 100 μL TEAB buffer (pH 8.5). Proteins were digested with trypsin overnight (1:40 trypsin: protein) at 37 °C. SDS was removed using Detergent Removal Columns (Pierce, Thermo Scientific, Rockford, IL, USA) per the manufacturer protocol.

Samples were labeled with a TMT 6plex reagents set (Thermo Scientific) according to the manufacturer instructions. Equal aliquots from each of the six TMT-tagged samples were pooled dried and fractionated by strong cation exchange using SCX Stage Tips (Affinisep, Le Houlme, France) then dried. Peptides were dissolved in 10 μL 0.1% formic acid for LC–MS analysis on a Dionex Ultimate 3000 RSLC system (Thermo Scientific) coupled to a Q-Exactive Orbitrap mass spectrometer (Thermo Scientific). The mobile phases were A: 0.1% formic acid in water; B: 0.1% formic acid in acetonitrile. Initial conditions were 5% B. Peptides (1 μg) were loaded onto a C18 PepMap 100 trap column (5 μm, 100 Å, 5 × 0.3 mm) at 20 μL/min with 2% B, then separated on an Acclaim PepMap 100 C18 analytical column (3 μm, 100 Å, 25 × 0.075 mm) at 0.3 μL/min using a gradient: hold at 5% B (7 min), increase to 10% B (8 min), increase to 30% B (128 min), increase to 60% B (135 min) increase to 95% B (136 min), hold at 95% B (141 min), return to 5% B (142 min), and equilibrate (5 min).

Nanoelectrospray ionization was performed with a 30 μm I.D. stainless-steel emitter at 1.9 kV. MS spectra were acquired in data-dependent acquisition mode (cycle of 10) using 70,000 resolution for MS and 35,000 for MS/MS, with a 1.4 *m*/*z* isolation window and normalized collision energy of 32. Raw files were analyzed using Proteome Discoverer v2.4 (Thermo Scientific) with SEQUEST HT and Mascot (Matrix Science, London, UK) search engines, against the UniProt *P. aeruginosa* reference proteomes (strain PAO1, proteome ID UP000002438).

### Metabolomics Analysis

Biofilm growth and cell pellet isolation was described above. To each cell pellet, 500 μL of methanol: water (1:1) containing 0.1% butylated hydroxytoluene (BHT) was added. Samples were vortexed for 30 s, sonicated 5 min in an ultrasonic bath, and placed on an ice bath (1 h) to precipitate proteins. After centrifugation (10,000*g*, 5 min), the supernatants were transferred to a new tube, mixed with 500 μL chloroform, and vortexed (10 min). The mixture was then centrifuged (15,000*g*, 5 min), the aqueous phase was collected, and the extraction repeated. Pooled aqueous phases were dried under a gentle stream of nitrogen and stored at −20 °C.

Mass spectrometry was performed on a Waters Synapt XS ESI-Q-IMS-TOF mass spectrometer coupled to a Waters Acquity Premiere UPLC system. Samples were analyzed with capillary voltages 1 kV (positive mode) and 2 kV (negative mode). The mass range monitored was 50–1200 *m*/*z*. Lock-mass calibration was performed using a Leucine-Enkephalin solution, infused every 10 s via a secondary, orthogonal sprayer. The instrument operated in MSE mode, with data acquired at 0.25 s per scan and a fragmentation ramp of 10–30 V. Chromatographic separation was carried out on a Waters Premiere BEH Amide column (150 × 2.7 mm, 1.7 μm pore size) using a binary gradient at a constant flow rate of 400 μL/min. Mobile phase composition was as follows: A = H_2_O with 0.1% formic acid, and B = acetonitrile with 0.1% formic acid. The gradient program was: 0–1 min, 5% B; 1–7 min, 95% B; 7–9 min, 95% B; 9–9.1 min 5% B; 9.1–11 min, 5% B. Data acquisition and initial inspection were performed using MassLynx (v4.2). Raw data files were imported into Progenesis QI (v3.1) for alignment, peak picking, and deconvolution with default parameters, except for high energy fragment alignment the retention time tolerance was set to 4 sampling intervals and chromatographic similarity was set to 0.7. Compound identification was initially performed against an in-built database of metabolite standards for which retention time and fragmentation profile were recorded on the same acquisition platform. IDs not matching this database were further searched using the METLIN 2019 MS/MS database. Remaining unassigned peaks were further searched using a theoretical fragmentation algorithm within Progenesis QI, against structure databases (HDMB for polar compounds). Only those candidate matches scoring at least two-thirds (40) of the maximum possible score of 60 were retained for further consideration. Compound assignments were based on the highest scoring match meeting this criterion.

### Measurement of Minimum Inhibitory Concentrations (MIC)

The MIC of antibiotics against three strains of *P. aeruginosa* PAO1 were determined as previously described,^27^ using the broth microdilution method according to Clinical and Laboratory Standard Institute (CLSI) guidelines,^73^ but culturing the cells in M63 media.

## Supplementary Material



## Notes

Tables S1–S4 listing proteins and metabolites exhibiting differential levels in KM-5-35-treated cells compared to untreated controls (PDF)
