# Supplementary material for: Inhibiting Bacterioferritin Iron Release Induces Iron Starvation and Metabolic Downshift, Potentiating Aminoglycosides in P. aeruginosa Biofilms
Source: ACS Infect Dis. 2026 Jun 15;12(7):2358–71. doi: 10.1021/acsinfecdis.6c00329 (PMC13366587; doi:10.1021/acsinfecdis.6c00329)
Supplement: Supplementary file 1 [file id6c00329_si_001.pdf]

SUPPORTING INFORMATION

Inhibiting Bacterioferritin Iron Release Induces Iron Starvation and Metabolic Downshift, Potentiating Aminoglycosides in *P. aeruginosa* Biofilms

*Leo Fontenot[1, §], Huili Yao[1, §], Alexanndra M. Behm[1], Anabel Soldano[1], Fabrizio Donnarumma[1], Richard A. Bunce[2], Mario Rivera[1 *]*

[1]Department of Chemistry, Louisiana State University, Baton Rouge, LA, 70803, USA

[2]Department of Chemistry, Oklahoma State Univeristy, Stillwater, OK 74078, USA.

[§]These authors contributed equally

*Corresponding author. Email: mrivera@lsu.edu

ORCID: 0000-0002-5692-5497

Table S1. Proteins that exhibit significant abundance differences between KM-5-35-treated *P. aeruginosa* cells and untreated controls.

Locus ID	Name	Product	Log_2FC (treated/unt)	*p* value
PA2315		hypothetical protein	1.58	1.33E-02
PA2272	pbpC	penicillin-binding protein 3A	1.33	5.04E-03
PA0312		conserved hypothetical protein	1.30	4.35E-03
PA2973		probable peptidase	1.24	5.91E-04
PA1288	odsT	probable outer membrane protein precursor	1.21	1.73E-02
PA2242	pslL	hypothetical protein	1.21	3.78E-02
PA5333		conserved hypothetical protein	1.20	5.31E-03
PA4886		probable two-component sensor	1.20	1.14E-03
PA5220		hypothetical protein	1.19	6.67E-04
PA3180		hypothetical protein	1.18	8.33E-03
PA0153	pcaH	protocatechuate 3,4-dioxygenase, beta subunit	1.15	3.84E-04
PA2565		hypothetical protein	1.14	1.32E-02
PA3235		conserved hypothetical protein	1.13	1.13E-02
PA2536		probable phosphatidate cytidylyltransferase	1.11	1.86E-02
PA0164		probable gamma-glutamyltranspeptidase	1.10	1.41E-02
PA4925		conserved hypothetical protein	1.09	3.66E-03
PA1627		probable transcriptional regulator	1.08	4.69E-02
PA2555		probable AMP-binding enzyme	1.06	8.76E-04
PA2533		probable sodium:alanine symporter	1.05	3.23E-02
PA4143		probable toxin transporter	1.05	5.62E-04
PA1323		hypothetical protein	1.04	2.89E-02
PA3041		hypothetical protein	1.03	1.06E-02
PA4212	phzC1	phenazine biosynthesis protein PhzC	1.03	1.52E-02
PA3425		hypothetical protein	1.03	1.86E-02
PA2020	mexZ	MexZ	1.03	1.12E-02
PA5276	lppL	Lipopeptide LppL precursor	1.03	9.66E-03
PA4811	fdnH	nitrate-inducible formate dehydrogenase, beta subunit	1.01	1.20E-02
PA5090	vgrG5	VgrG5	1.01	6.37E-03
PA0341	lgt	prolipoprotein diacylglyceryl transferase	0.99	2.14E-02
PA3373		conserved hypothetical protein	0.98	1.83E-03
PA0730		probable transferase	0.98	1.63E-02
PA3133	sawR	SawR	0.98	8.73E-04
PA3233		hypothetical protein	0.97	2.04E-03
PA5147	mutY	A / G specific adenine glycosylase	0.97	1.66E-02
PA0256		hypothetical protein	0.97	5.31E-03
PA0178		probable two-component sensor	0.96	1.33E-02
PA4809	fdhE	FdhE protein	0.95	4.46E-03
PA0538	dsbB	disulfide bond formation protein	0.95	2.28E-02

PA3770	guaB	inosine-5'-monophosphate dehydrogenase	0.94	4.15E-03
PA1044		hypothetical protein	0.94	1.52E-03
PA4810	fdnI	nitrate-inducible formate dehydrogenase, gamma subunit	0.94	3.66E-02
PA4728	folK	2-amino-4-hydroxy-6-hydroxymethyldihydropteridine pyrophosphokinase	0.94	2.07E-02
PA0736		hypothetical protein	0.93	6.69E-03
PA2173		hypothetical protein	0.93	3.89E-02
PA0414	chpB	probable methylesterase	0.93	4.15E-02
PA1396		probable two-component sensor	0.93	6.38E-03
PA3091		hypothetical protein	0.93	6.01E-04
PA1092	fliC	flagellin type B	0.92	2.50E-03
PA5143	hisB	imidazoleglycerol-phosphate dehydratase	0.92	7.13E-03
PA4029		conserved hypothetical protein	0.92	6.64E-04
PA4351	olsA	OlsA	0.92	5.35E-03
PA3822		conserved hypothetical protein	0.90	1.68E-04
PA4557	lytB	LytB protein	0.90	1.96E-02
PA5154		probable permease of ABC transporter	0.90	3.12E-02
PA2267		probable transcriptional regulator	0.89	1.38E-02
PA1872		hypothetical protein	0.88	7.01E-04
PA3189	gltF	probable permease of ABC sugar transporter	0.88	1.78E-02
PA4385	groEL	GroEL protein	0.87	9.33E-03
PA2365	hsiB3	HsiB3	0.87	1.51E-02
PA0410	pilI	twitching motility protein PilI	0.87	1.11E-03
PA5061		conserved hypothetical protein	0.86	2.03E-02
PA4487	magF	MagF	0.85	2.09E-03
PA4454		conserved hypothetical protein	0.84	3.91E-02
PA3641		probable amino acid permease	0.83	3.45E-03
PA4845	dipZ	thiol:disulfide interchange protein DipZ	0.83	8.73E-03
PA4431		probable iron-sulfur protein	0.82	2.02E-02
PA1553	ccoO1	Cytochrome c oxidase, cbb3-type, CcoO subunit	0.82	2.59E-03
PA4931	dnaB	replicative DNA helicase	0.82	1.33E-02
PA0118		hypothetical protein	0.82	4.32E-03
PA2713		conserved hypothetical protein	0.82	1.81E-02
PA1523	xdhB	xanthine dehydrogenase	0.82	3.30E-02
PA5119	glnA	glutamine synthetase	0.81	2.23E-02
PA2493	mexE	Resistance-Nodulation-Cell Division (RND) multidrug efflux membrane fusion protein MexE precursor	0.81	3.88E-04
PA0934	relA	GTP pyrophosphokinase	0.81	7.22E-04
PA4525	pilA	type 4 fimbrial precursor PilA	0.80	1.90E-02
PA3221	csaA	CsaA protein	0.80	2.83E-02
PA1432	lasI	autoinducer synthesis protein LasI	0.80	6.16E-03
PA2705		hypothetical protein	0.80	1.27E-03
PA0919		alanyl-phosphatidylglycerol hydrolase	0.80	1.00E-02

PA1465		hypothetical protein	0.80	2.00E-02
PA1591		hypothetical protein	0.80	4.66E-02
PA3082	gbt	glycine betaine transmethylase	0.79	8.20E-03
PA2696		probable transcriptional regulator	0.79	6.84E-03
PA0280	cysA	sulfate transport protein CysA	0.79	1.20E-03
PA3204	cpxR	two-component response regulator CpxR	0.79	1.06E-02
PA5017	dipA	DipA	0.79	3.63E-03
PA3486	vgrG4b	VgrG4b	0.79	1.71E-02
PA0858		conserved hypothetical protein	0.79	2.67E-02
PA0426	mexB	Resistance-Nodulation-Cell Division (RND) multidrug efflux transporter MexB	0.78	6.57E-04
PA3820	secF	secretion protein SecF	0.78	1.15E-02
PA4131		probable iron-sulfur protein	0.78	1.09E-02
PA0622		probable bacteriophage protein	0.78	2.03E-03
PA1598		conserved hypothetical protein	0.78	8.51E-03
PA2240	pslJ	PslJ	0.78	2.31E-02
PA2151		conserved hypothetical protein	0.77	7.08E-03
PA5397		hypothetical protein	0.77	3.64E-03
PA2373	vgrG3	VgrG3	0.77	3.62E-02
PA3311	nbdA	NbdA	0.77	1.11E-02
PA5438		probable transcriptional regulator	0.77	8.62E-03
PA3697		hypothetical protein	0.76	1.37E-03
PA4689		hypothetical protein	0.76	4.13E-03
PA2527	muxB	MuxB	0.76	1.29E-03
PA4167		probable oxidoreductase	0.76	4.72E-02
PA3042		hypothetical protein	0.76	2.48E-03
PA1446	fliP	flagellar biosynthetic protein FliP	0.76	3.16E-02
PA5077	opgH	OpgH	0.75	2.88E-03
PA3905	tecT	type VI effector chaperone for Tox-Rease, TecT	0.75	4.88E-03
PA4145		probable transcriptional regulator	0.75	4.15E-03
PA2118	ada	O6-methylguanine-DNA methyltransferase	0.75	1.52E-02
PA1585	sucA	2-oxoglutarate dehydrogenase (E1 subunit)	0.74	8.77E-04
PA2991	sth	soluble pyridine nucleotide transhydrogenase	0.74	2.46E-03
PA2005	hbcR	HbcR	0.74	4.54E-03
PA5255	algQ	Alginate regulatory protein AlgQ	0.74	3.34E-02
PA0051	phzH	potential phenazine-modifying enzyme	0.74	2.82E-02
PA4778	cueR	CueR	0.74	9.94E-03
PA0357	mutM	formamidopyrimidine-DNA glycosylase	0.74	1.93E-02
PA4280	birA	BirA bifunctional protein	0.74	1.41E-03
PA1484		probable transcriptional regulator	0.74	4.18E-02
PA3707	wspB	hypothetical protein	0.74	3.40E-02
PA3479	rhlA	rhamnosyltransferase chain A	0.73	4.12E-02

PA1686	alkA	DNA-3-methyladenine glycosidase II	0.73	4.54E-02
PA1741		hypothetical protein	0.73	4.91E-02
PA2722		hypothetical protein	0.73	4.51E-02
PA4790		conserved hypothetical protein	0.73	2.12E-03
PA3790	oprC	Putative copper transport outer membrane porin OprC precursor	0.72	1.78E-02
PA4067	oprG	Outer membrane protein OprG precursor	0.72	1.38E-03
PA1641		hypothetical protein	0.72	5.69E-03
PA4669	ipk	isopentenyl monophosphate kinase	0.72	7.30E-03
PA2523	czcR	CzcR	0.72	2.02E-03
PA3626		conserved hypothetical protein	0.72	1.15E-02
PA2360	hsiA3	hypothetical protein	0.72	7.53E-03
PA5475		hypothetical protein	0.72	2.33E-02
PA3084		hypothetical protein	0.72	1.19E-03
PA2617	aat	leucyl/phenylalanyl-tRNA-protein transferase	0.72	2.02E-02
PA2406	fpvK	FpvK	0.72	2.63E-02
PA3040		conserved hypothetical protein	0.71	1.25E-02
PA4606		conserved hypothetical protein	0.71	4.80E-05
PA0158	triC	Resistance-Nodulation-Cell Division (RND) triclosan efflux transporter, TriC	0.71	1.76E-03
PA2586	gacA	response regulator GacA	0.71	7.77E-03
PA4078		probable nonribosomal peptide synthetase	0.71	1.96E-03
PA3816	cysE	O-acetylserine synthase	0.71	4.19E-03
PA2948	cobM	precorrin-3 methylase	0.71	8.61E-04
PA4655	hemH	ferrochelatase	0.71	9.47E-03
PA1165	pcpS	PcpS	0.71	4.90E-03
PA2150		conserved hypothetical protein	0.71	4.44E-02
PA0203		probable binding protein component of ABC transporter	0.71	1.90E-02
PA2291		probable glucose-sensitive porin	0.70	4.17E-02
PA0396	pilU	twitching motility protein PilU	0.70	2.24E-03
PA5107	blc	outer membrane lipoprotein Blc	0.70	8.88E-04
PA0196	pntB	pyridine nucleotide transhydrogenase, beta subunit	0.70	1.93E-04
PA3680		conserved hypothetical protein	0.70	1.67E-02
PA0265	davD	glutaric semialdehyde dehydrogenase	0.69	3.55E-03
PA0958	oprD	Basic amino acid, basic peptide and imipenem outer membrane porin OprD precursor	0.69	2.52E-02
PA1494	muiA	mucoidy inhibitor gene A	0.69	7.67E-03
PA4456		probable ATP-binding component of ABC transporter	0.69	8.90E-03
PA0359		hypothetical protein	0.69	2.34E-02
PA3515		hypothetical protein	0.69	2.29E-02
PA4354		conserved hypothetical protein	0.69	3.66E-03
PA0391		hypothetical protein	0.69	2.50E-03
PA0491		probable transcriptional regulator	0.69	1.27E-02
PA3068	gdhB	NAD-dependent glutamate dehydrogenase	0.68	2.91E-04

PA4310	pctB	chemotactic transducer PctB	0.68	4.95E-03
PA4760	dnaJ	DnaJ protein	0.68	1.29E-03
PA3271		probable two-component sensor	0.68	1.36E-02
PA5428		probable transcriptional regulator	0.68	2.53E-04
PA1667	hsiJ2	HsiJ2	0.68	1.89E-02
PA5039	aroK	shikimate kinase	0.68	7.09E-03
PA3147	wbpJ	probable glycosyl transferase WbpJ	0.68	4.10E-03
PA1009		hypothetical protein	0.68	2.90E-02
PA2947	cobE	CobE	0.68	1.45E-02
PA1899	phzA2	probable phenazine biosynthesis protein	0.68	4.93E-02
PA0217		probable transcriptional regulator	0.68	6.39E-03
PA4780		conserved hypothetical protein	0.68	5.69E-03
PA2459		hypothetical protein	0.68	1.47E-02
PA2815	yafH	probable acyl-CoA dehydrogenase	0.67	1.63E-02
PA3727		hypothetical protein	0.67	8.30E-03
PA0783	putP	sodium/proline symporter PutP	0.67	7.76E-03
PA0535		probable transcriptional regulator	0.67	2.24E-03
PA0455	dbpA	RNA helicase DbpA	0.67	1.65E-02
PA4656		conserved hypothetical protein	0.67	2.33E-02
PA2810	copS	two-component sensor, CopS	0.67	1.57E-02
PA1637	kdpE	two-component response regulator KdpE	0.67	2.08E-04
PA1885		conserved hypothetical protein	0.67	4.83E-02
PA1832		probable protease	0.66	2.12E-03
PA1657	hsiB2	HsiB2	0.66	2.90E-02
PA0156	triA	Resistance-Nodulation-Cell Division (RND) triclosan efflux membrane fusion protein, TriA	0.66	2.33E-03
PA0527	dnr	transcriptional regulator Dnr	0.66	1.14E-02
PA1315		probable transcriptional regulator	0.66	1.26E-02
PA3199		conserved hypothetical protein	0.66	7.59E-03
PA2043		hypothetical protein	0.66	3.35E-02
PA0425	mexA	Resistance-Nodulation-Cell Division (RND) multidrug efflux membrane fusion protein MexA precursor	0.65	2.57E-03
PA4876	osmE	osmotically inducible lipoprotein OsmE	0.65	1.20E-02
PA4430		probable cytochrome b	0.65	1.33E-03
PA5268	corA	magnesium/cobalt transport protein	0.65	4.09E-03
PA3344	recQ	ATP-dependent DNA helicase RecQ	0.65	7.20E-03
PA0236		probable transcriptional regulator	0.65	3.88E-03
PA1658	hsiC2	HsiC2	0.64	3.71E-02
PA0262	vgrG2b	VgrG2b	0.64	3.04E-03
PA1527		conserved hypothetical protein	0.64	4.70E-03
PA3934		conserved hypothetical protein	0.64	2.93E-02
PA4350	olsB	OlsB	0.64	2.88E-02
PA1136		probable transcriptional regulator	0.64	6.65E-03

PA3575		hypothetical protein	0.64	4.59E-02
PA1960		hypothetical protein	0.64	4.92E-02
PA1161	rrmA	rRNA methyltransferase	0.64	4.68E-02
PA3729		conserved hypothetical protein	0.63	6.93E-03
PA3349		probable chemotaxis protein	0.63	5.22E-03
PA3348	cheR1	CheR1	0.63	8.14E-03
PA0756		probable two-component response regulator	0.63	5.23E-04
PA0428		probable ATP-dependent RNA helicase	0.63	1.01E-03
PA3620	mutS	DNA mismatch repair protein MutS	0.63	9.83E-03
PA2901		hypothetical protein	0.63	6.05E-03
PA1760		probable transcriptional regulator	0.63	2.62E-02
PA3599		probable transcriptional regulator	0.63	1.35E-02
PA3980		conserved hypothetical protein	0.63	2.49E-02
PA4064		probable ATP-binding component of ABC transporter	0.63	2.19E-02
PA3321		probable transcriptional regulator	0.63	7.04E-03
PA0422		conserved hypothetical protein	0.63	9.88E-03
PA2233	pslC	PslC	0.63	2.64E-02
PA1128		probable transcriptional regulator	0.63	4.23E-02
PA3259		hypothetical protein	0.63	3.11E-02
PA5232		conserved hypothetical protein	0.62	1.26E-02
PA2561	ctpH	CtpH	0.62	2.94E-03
PA5558	atpF	ATP synthase B chain	0.62	5.18E-03
PA4732	pgi	glucose-6-phosphate isomerase	0.62	1.03E-02
PA0291	oprE	Anaerobically-induced outer membrane porin OprE precursor	0.62	6.90E-04
PA0001	dnaA	chromosomal replication initiator protein DnaA	0.62	6.50E-03
PA5490	cc4	cytochrome c4 precursor	0.62	4.10E-02
PA4565	proB	glutamate 5-kinase	0.62	1.69E-03
PA1822	fimL	hypothetical protein	0.62	7.16E-04
PA3806		conserved hypothetical protein	0.62	1.79E-02
PA1125		probable cobalamin biosynthetic protein	0.62	1.79E-02
PA1640		conserved hypothetical protein	0.62	3.70E-03
PA2495	oprN	Multidrug efflux outer membrane protein OprN precursor	0.61	6.51E-03
PA5146		hypothetical protein	0.61	5.79E-04
PA4257	rpsC	30S ribosomal protein S3	0.61	5.58E-03
PA2830	htpX	heat shock protein HtpX	0.61	2.25E-02
PA3984	lnt	apolipoprotein N-acyltransferase	0.61	2.06E-03
PA4556	pilE	type 4 fimbrial biogenesis protein PilE	0.61	4.11E-02
PA3291	tli1	Tli1	0.61	3.19E-02
PA4919	pncB1	nicotinate phosphoribosyltransferase	0.60	2.27E-04
PA4812	fdnG	| formate dehydrogenase-O, major subunit	0.60	6.72E-04
PA4959	fimX	FimX	0.60	2.99E-03
PA0652	vfr	transcriptional regulator Vfr	0.60	1.03E-03

PA0867	mliC	membrane-bound lysozyme inhibitor of c-type lysozyme MliC	0.60	4.03E-03
PA3452	mqoA	malate:quinone oxidoreductase	0.60	5.18E-04
PA2237	pslG	PslG	0.60	2.09E-02
PA0356		hypothetical protein	0.60	1.13E-03
PA1996	ppiC1	peptidyl-prolyl cis-trans isomerase C1	0.60	2.99E-02
PA2649	nuoN	NADH dehydrogenase I chain N	0.60	7.11E-03
PA0352		probable transporter	0.60	1.87E-02
PA2775	tsi4	Tsi4	0.60	5.23E-03
PA2076	otsR	probable transcriptional regulator	0.60	1.09E-02
PA5027		hypothetical protein	0.60	5.24E-04
PA1488		hypothetical protein	0.60	2.97E-02
PA3821	secD	secretion protein SecD	0.59	5.26E-04
PA2881		probable two-component response regulator	0.59	2.00E-02
PA2232	pslB	PslB	0.59	1.50E-02
PA4426		conserved hypothetical protein	0.59	1.40E-03
PA1324		hypothetical protein	0.59	6.64E-03
PA4961		hypothetical protein	0.59	5.70E-05
PA1775	cmpX	conserved cytoplasmic membrane protein, CmpX protein	0.59	2.84E-02
PA3301		hypothetical protein	0.59	3.10E-03
PA3948	rocA1	Two-component response regulator RocA1	0.59	7.06E-03
PA2557		probable AMP-binding enzyme	0.59	4.87E-03
PA3212		probable ATP-binding component of ABC transporter	0.59	1.20E-02
PA4024	eutB	ethanolamine-ammonia lyase, large subunit, EutB	0.59	4.75E-02
PA2445	gcvP2	glycine cleavage system protein P2	0.58	1.52E-02
PA2494	mexF	Resistance-Nodulation-Cell Division (RND) multidrug efflux transporter MexF	0.58	1.73E-02
PA2953		electron transfer flavoprotein-ubiquinone oxidoreductase	0.58	2.13E-02
PA4942	hflK	protease subunit HflK	0.58	1.49E-02
PA4209	phzM	probable phenazine-specific methyltransferase	0.58	4.65E-03
PA5213	gcvP1	glycine cleavage system protein P1	0.58	3.20E-02
PA3636	kdsA	2-dehydro-3-deoxyphosphooctonate aldolase	0.58	9.78E-03
PA0401		noncatalytic dihydroorotase-like protein	0.58	2.10E-02
PA3461		conserved hypothetical protein	0.58	3.09E-02
PA3460		probable acetyltransferase	0.58	3.58E-02
PA1397		probable two-component response regulator	0.58	8.68E-04
PA1791		hypothetical protein	0.58	2.56E-03
PA4133		cytochrome c oxidase subunit (cbb3-type)	0.58	3.76E-02
PA2660		hypothetical protein	0.58	2.53E-02
PA2392	pvdP	PvdP	0.58	3.97E-02
PA0074	ppkA	serine/threonine protein kinase PpkA	0.58	1.79E-02
PA4998		conserved hypothetical protein	0.58	3.94E-02
PA0011	htrB1	acyltransferase HtrB1	0.58	1.17E-02

PA5492		conserved hypothetical protein	0.58	6.81E-03
PA4954	motA	chemotaxis protein MotA	0.58	6.64E-03
PA2327		probable permease of ABC transporter	0.58	2.37E-02
PA4353		conserved hypothetical protein	0.58	8.50E-04
PA3794		hypothetical protein	0.58	3.82E-02
PA0439		probable oxidoreductase	0.58	4.07E-02
PA5455		putative glycosyltransferase	0.58	3.01E-02
PA4108		cyclic di-GMP phosphodiesterase	0.58	1.71E-02
PA4051	thiL	thiamine monophosphate kinase	0.58	3.61E-02
PA4751	ftsH	cell division protein FtsH	0.57	1.82E-03
PA2399	pvdD	pyoverdine synthetase D	0.57	3.62E-03
PA1818	cadA	lysine decarboxylase	0.57	1.72E-02
PA2945		conserved hypothetical protein	0.57	1.37E-02
PA2345		conserved hypothetical protein	0.57	1.37E-02
PA4235	ftnA	bacterial ferritin	0.57	5.20E-03
PA5065	ubiB	ubiquinone biosynthetic protein UbiB	0.57	1.93E-02
PA1293		hypothetical protein	0.57	6.66E-03
PA4374	mexV	Resistance-Nodulation-Cell Division (RND) multidrug efflux membrane fusion protein MexV	0.57	1.27E-02
PA2535		probable oxidoreductase	0.57	9.69E-05
PA5316	rpmB	50S ribosomal protein L28	0.57	1.51E-02
PA4019	ubiX	UbiX	0.57	1.13E-02
PA5279		conserved hypothetical protein	0.57	4.52E-03
PA4269	rpoC	DNA-directed RNA polymerase beta* chain	0.56	1.10E-03
PA4489	magD	MagD	0.56	6.62E-04
PA3848		hypothetical protein	0.56	3.42E-03
PA4785		probable acyl-CoA thiolase	0.56	8.94E-03
PA4633		probable chemotaxis transducer	0.56	3.72E-03
PA0095	vgrG1b	vgrG1b	0.56	1.21E-02
PA3335		hypothetical protein	0.56	3.42E-02
PA2827		conserved hypothetical protein	0.56	2.80E-02
PA4438		conserved hypothetical protein	0.56	1.17E-02
PA5115		conserved hypothetical protein	0.56	3.70E-04
PA3226		probable hydrolase	0.56	3.48E-04
PA4953	motB	chemotaxis protein MotB	0.56	3.18E-02
PA4949		conserved hypothetical protein	0.56	8.46E-03
PA3526	motY	MotY	0.56	3.60E-02
PA2271		probable acetyltransferase	0.56	3.25E-03
PA5554	atpD	ATP synthase beta chain	0.55	5.74E-04
PA2402	pvdI	probable non-ribosomal peptide synthetase	0.55	1.18E-02
PA3333	fabH2	3-oxoacyl-[acyl-carrier-protein] synthase III	0.55	2.21E-02
PA1097	fleQ	transcriptional regulator FleQ	0.55	1.44E-03

PA5557	atpH	ATP synthase delta chain	0.55	1.14E-02
PA3860		probable AMP-binding enzyme	0.55	2.02E-02
PA2022		probable nucleotide sugar dehydrogenase	0.55	3.32E-02
PA3213		hypothetical protein	0.55	1.09E-02
PA0715		hypothetical protein	0.55	5.05E-03
PA3050	pyrD	dihydroorotate dehydrogenase	0.55	1.60E-02
PA1256	lhpO	ABC transporter ATP-binding protein, LhpO	0.55	4.39E-03
PA4268	rpsL	30S ribosomal protein S12	0.55	3.15E-02
PA2727		hypothetical protein	0.55	3.72E-02
PA1511	vgrG2a	VgrG2a	0.55	1.12E-02
PA2961	holB	DNA polymerase III, delta prime subunit	0.55	2.13E-02
PA0608		probable phosphoglycolate phosphatase	0.55	3.71E-02
PA2332		probable transcriptional regulator	0.55	1.51E-02
PA4686	mksB	hypothetical protein	0.55	2.91E-02
PA1521		probable guanine deaminase	0.55	2.48E-02
PA4309	pctA	chemotactic transducer PctA	0.54	7.52E-04
PA2305	ambB	AmbB	0.54	1.12E-02
PA0833		hypothetical protein	0.54	3.93E-03
PA2014	liuB	methylcrotonyl-CoA carboxylase, beta-subunit	0.54	8.78E-03
PA2398	fpvA	ferripyoverdine receptor	0.54	7.93E-03
PA2652		methyl-accepting chemotaxis protein	0.54	1.37E-03
PA1169		probable lipoxygenase	0.54	7.08E-04
PA2882		probable two-component sensor	0.54	3.40E-03
PA4749	glmM	phosphoglucosamine mutase	0.54	3.58E-02
PA3944		conserved hypothetical protein	0.54	2.76E-03
PA0577	dnaG	DNA primase	0.54	4.34E-02
PA5296	rep	ATP-dependent DNA helicase Rep	0.54	2.92E-02
PA1731		conserved hypothetical protein	0.54	4.00E-03
PA0504	bioD	dethiobiotin synthase	0.54	5.48E-03
PA4004		conserved hypothetical protein	0.54	4.86E-03
PA3602		conserved hypothetical protein	0.53	6.33E-03
PA3535		probable serine protease	0.53	9.07E-05
PA3378		conserved hypothetical protein	0.53	1.62E-02
PA4476		hypothetical protein	0.53	1.43E-03
PA0079	tssK1	TssK1	0.53	2.72E-03
PA0735		hypothetical protein	0.53	1.02E-02
PA4787		probable transcriptional regulator	0.53	4.19E-04
PA4455	mlaE	probable permease of ABC transporter	0.53	1.83E-02
PA0657		probable ATPase	0.53	1.40E-02
PA1563	ygdE	conserved hypothetical protein	0.53	3.40E-02
PA0338		hypothetical protein	0.53	2.37E-02
PA4284	recB	exodeoxyribonuclease V beta chain	0.53	4.13E-02

PA5417	soxD	sarcosine oxidase delta subunit	0.53	5.00E-02
PA2424	pvdL	PvdL	0.52	1.59E-02
PA2540		conserved hypothetical protein	0.52	5.23E-03
PA4367	bifA	BifA	0.52	5.57E-03
PA3238		hypothetical protein	0.52	1.03E-03
PA4604		conserved hypothetical protein	0.52	9.32E-03
PA5043	pilN	type 4 fimbrial biogenesis protein PilN	0.52	1.31E-02
PA4501	opdP	Glycine-glutamate dipeptide porin OpdP	0.52	1.21E-02
PA0541		hypothetical protein	0.52	2.48E-03
PA4381	colR	two-component response regulator ColR	0.52	4.01E-02
PA2993		conserved hypothetical protein	0.52	9.89E-03
PA1341	aatQ	AatQ	0.52	2.66E-02
PA2256	pvcC	paerucumarin biosynthesis protein PvcC	0.52	3.19E-03
PA1615		probable lipase	0.52	1.35E-02
PA4798		hypothetical protein	0.52	3.54E-02
PA4494	roxS	RoxS	0.52	2.78E-03
PA2002		conserved hypothetical protein	0.52	4.23E-03
PA2316		probable transcriptional regulator	0.52	1.60E-02
PA2122		hypothetical protein	0.52	2.40E-02
PA0965	ruvC	Holliday junction resolvase RuvC	0.52	3.81E-02
PA0084	tssC1	TssC1	0.51	3.60E-02
PA4941	hflC	protease subunit HflC	0.51	1.48E-04
PA2400	pvdJ	PvdJ	0.51	3.12E-02
PA1032	quiP	QuiP	0.51	1.60E-03
PA5210		probable secretion pathway ATPase	0.51	1.08E-02
PA2857		probable ATP-binding component of ABC transporter	0.51	1.12E-03
PA5231		probable ATP-binding/permease fusion ABC transporter	0.51	4.68E-03
PA4538	ndh	NADH dehydrogenase	0.51	3.51E-02
PA4825	mgtA	Mg(2+) transport ATPase, P-type 2	0.51	3.25E-02
PA2728		hypothetical protein	0.51	7.06E-03
PA4411	murC	UDP-N-acetylmuramate--alanine ligase	0.51	4.01E-03
PA2840		probable ATP-dependent RNA helicase	0.51	2.46E-03
PA3883		probable short-chain dehydrogenase	0.51	2.01E-02
PA0082	tssA1	TssA1	0.51	3.46E-03
PA1290		probable transcriptional regulator	0.51	4.94E-02
PA5556	atpA	ATP synthase alpha chain	0.50	1.18E-03
PA3280	oprO	Pyrophosphate-specific outer membrane porin OprO precursor	0.50	7.40E-03
PA2788		probable chemotaxis transducer	0.50	2.89E-02
PA2367	hcp3	Hcp3	0.50	1.70E-02
PA3150	wpbG	LPS biosynthesis protein WbpG	0.50	2.55E-02
PA0892	aotP	arginine/ornithine transport protein AotP	0.50	1.06E-02
PA3380		conserved hypothetical protein	0.50	7.30E-03

PA4362		hypothetical protein	0.50	3.15E-02
PA4915		probable chemotaxis transducer	0.50	5.42E-03
PA4727	pcnB	poly(A) polymerase	0.50	2.38E-03
PA0920		alanyl-phosphatidylglycerol synthase	0.50	1.15E-02
PA3468		conserved hypothetical protein	0.50	4.11E-04
PA1045		hypothetical protein	0.50	1.54E-03
PA1487		probable carbohydrate kinase	0.50	5.18E-03
PA5163	rmlA	glucose-1-phosphate thymidylyltransferase	-0.50	2.98E-02
PA4854	purH	phosphoribosylaminoimidazolecarboxamide formyltransferase	-0.50	2.38E-02
PA0902		hypothetical protein	-0.50	6.04E-03
PA2352		probable glycerophosphoryl diester phosphodiesterase	-0.50	7.72E-03
PA4336		conserved hypothetical protein	-0.50	1.43E-02
PA5141	hisA	phosphoribosylformimino-5-aminoimidazole carboxamide	-0.50	1.12E-02
PA1689		conserved hypothetical protein	-0.50	9.63E-03
PA4930	alr	biosynthetic alanine racemase	-0.50	2.29E-02
PA0954		probable acylphosphatase	-0.50	2.75E-02
PA3792	leuA	2-isopropylmalate synthase	-0.51	1.26E-02
PA4572	fklB	peptidyl-prolyl cis-trans isomerase FklB	-0.51	3.28E-02
PA3181		2-keto-3-deoxy-6-phosphogluconate aldolase	-0.51	1.05E-02
PA3173		probable short-chain dehydrogenase	-0.51	5.36E-03
PA3664		conserved hypothetical protein	-0.51	3.41E-02
PA0550		conserved hypothetical protein	-0.51	1.42E-02
PA0403	pyrR	transcriptional regulator PyrR	-0.51	2.36E-02
PA2982		conserved hypothetical protein	-0.51	7.43E-03
PA4744	infB	translation initiation factor IF-2	-0.52	7.63E-04
PA4017		conserved hypothetical protein	-0.52	8.95E-03
PA0105	coxB	cytochrome c oxidase, subunit II	-0.52	2.28E-02
PA5336	gmk	guanylate kinase	-0.52	3.28E-03
PA0266	davT	delta-aminovalerate aminotransferase	-0.53	2.14E-02
PA3987	leuS	leucyl-tRNA synthetase	-0.53	1.01E-02
PA0956	proS	prolyl-tRNA synthetase	-0.53	9.88E-03
PA3195	gapA	glyceraldehyde 3-phosphate dehydrogenase	-0.53	4.06E-02
PA4907		probable short-chain dehydrogenase	-0.53	6.66E-03
PA0430	metF	5,10-methylenetetrahydrofolate reductase	-0.53	1.52E-02
PA4932	rplI	50S ribosomal protein L9	-0.53	1.01E-02
PA5176		conserved hypothetical protein	-0.53	4.38E-03
PA3859		carboxylesterase	-0.53	2.00E-02
PA0388		hypothetical protein	-0.53	1.81E-02
PA0182		probable short-chain dehydrogenase	-0.53	4.96E-02
PA1307		conserved hypothetical protein	-0.53	2.73E-02
PA2010	hmgR	HmgR	-0.53	3.52E-02
PA2743	infC	translation initiation factor IF-3	-0.54	9.88E-03

PA5215	gcvT1	glycine-cleavage system protein T1	-0.54	1.27E-02
PA1203		hypothetical protein	-0.54	1.18E-03
PA4244	rplO	50S ribosomal protein L15	-0.54	4.69E-04
PA4475		conserved hypothetical protein	-0.54	1.57E-02
PA2385	pvdQ	3-oxo-C12-homoserine lactone acylase PvdQ	-0.54	1.83E-03
PA4702		hypothetical protein	-0.54	1.72E-02
PA1096		hypothetical protein	-0.54	4.74E-02
PA5357		hypothetical protein	-0.54	2.06E-02
PA4356	xenB	xenobiotic reductase	-0.55	7.43E-04
PA0870	phhc	aromatic amino acid aminotransferase	-0.55	1.04E-02
PA5173	arcC	carbamate kinase	-0.55	1.17E-02
PA2612	serS	seryl-tRNA synthetase	-0.55	1.61E-02
PA0537		conserved hypothetical protein	-0.55	1.56E-03
PA4465		conserved hypothetical protein	-0.55	3.80E-03
PA4564		conserved hypothetical protein	-0.55	5.28E-03
PA0741		conserved hypothetical protein	-0.55	1.66E-02
PA4217	phzS	flavin-containing monooxygenase	-0.55	1.22E-03
PA2625		conserved hypothetical protein	-0.55	4.71E-02
PA1234		hypothetical protein	-0.55	1.33E-02
PA3763	purL	phosphoribosylformylglycinamidine synthase	-0.56	4.43E-03
PA5322	algC	phosphomannomutase AlgC	-0.56	5.66E-03
PA2831		conserved hypothetical protein	-0.56	1.44E-03
PA1005		conserved hypothetical protein	-0.56	3.16E-03
PA3537	argF	ornithine carbamoyltransferase, anabolic	-0.56	3.23E-02
PA3574.1		zed protein	-0.56	1.43E-02
PA1927	metE	5-methyltetrahydropteroyltriglutamate-homocysteine S-methyltransferase	-0.57	2.36E-03
PA0506	fadE	probable acyl-CoA dehydrogenase	-0.57	6.51E-03
PA0007		hypothetical protein	-0.57	3.25E-03
PA0565		conserved hypothetical protein	-0.57	3.81E-02
PA1254	lhpC	delta1-pyrroline-4-hydroxy-2-carboxylate deaminase, LphC	-0.57	2.71E-02
PA3566		conserved hypothetical protein	-0.57	1.95E-02
PA1597		hypothetical protein	-0.57	4.98E-03
PA4497	dppA2	probable binding protein component of ABC transporter	-0.57	7.16E-03
PA5038	aroB	3-dehydroquinate synthase	-0.58	1.29E-02
PA3846		hypothetical protein	-0.58	1.34E-02
PA5260	hemC	porphobilinogen deaminase	-0.58	4.66E-03
PA3759		probable aminotransferase	-0.58	9.32E-04
PA3240		conserved hypothetical protein	-0.58	2.33E-03
PA0803		hypothetical protein	-0.58	7.29E-03
PA1726	bglX	periplasmic beta-glucosidase	-0.58	2.89E-03
PA0333		hypothetical protein	-0.58	4.13E-02

PA3190		probable binding protein component of ABC sugar transporter	-0.59	5.61E-03
PA1750		phospho-2-dehydro-3-deoxyheptonate aldolase	-0.59	1.90E-04
PA4626	hprA	glycerate dehydrogenase	-0.59	8.18E-04
PA4204	ppgL	periplasmic gluconolactonase, PpgL	-0.59	3.45E-03
PA3236	betX	BetX	-0.59	5.84E-03
PA3670		hypothetical protein	-0.59	3.10E-03
PA3110		hypothetical protein	-0.59	3.00E-02
PA2721		hypothetical protein	-0.59	1.14E-02
PA0055		hypothetical protein	-0.60	3.68E-02
PA1810	nppA2	NppA2	-0.60	1.91E-02
PA2902		hypothetical protein	-0.60	1.09E-02
PA4687	hitA	ferric iron-binding periplasmic protein HitA	-0.60	3.48E-02
PA1589	sucD	succinyl-CoA synthetase alpha chain	-0.61	3.83E-03
PA4602	glyA3	serine hydroxymethyltransferase	-0.61	1.02E-02
PA0143	nuh	purine nucleosidase Nuh	-0.61	4.19E-02
PA0371		hypothetical protein	-0.61	1.85E-02
PA1013	purC	phosphoribosylaminoimidazole-succinocarboxamide synthase	-0.61	1.18E-02
PA5562	spoOJ	chromosome partitioning protein Spo0J	-0.61	2.11E-03
PA5100	hutU	urocanase	-0.61	3.76E-03
PA4425		sedoheptulose 7-phosphate isomerase GmhA	-0.61	3.10E-02
PA4519	speC	ornithine decarboxylase	-0.62	1.13E-02
PA5441		hypothetical protein	-0.62	4.11E-03
PA5422		hypothetical protein	-0.62	1.27E-04
PA5521		probable short-chain dehydrogenase	-0.63	1.63E-03
PA4855	purD	phosphoribosylamine--glycine ligase	-0.63	4.69E-03
PA2331		hypothetical protein	-0.63	3.96E-02
PA1171	sltB2	SltB2	-0.63	3.75E-02
PA5395		conserved hypothetical protein	-0.63	2.00E-04
PA3260		probable transcriptional regulator	-0.63	4.68E-02
PA4402	argJ	glutamate N-acetyltransferase	-0.64	1.57E-04
PA4524	nadC	nicotinate-nucleotide pyrophosphorylase	-0.64	1.99E-03
PA3440.1		zed protein	-0.64	7.83E-04
PA0536		hypothetical protein	-0.64	3.23E-02
PA4498	mdpA	metallo-dipeptidase aeruginosa, MdpA	-0.64	2.50E-02
PA2117		hypothetical protein	-0.64	6.63E-04
PA4502	dppA4	probable binding protein component of ABC transporter	-0.64	4.71E-02
PA0764	mucB	negative regulator for alginate biosynthesis MucB	-0.65	5.84E-03
PA3879	narL	two-component response regulator NarL	-0.65	1.87E-02
PA3053		probable hydrolytic enzyme	-0.65	6.32E-04
PA1033		probable glutathione S-transferase	-0.65	5.18E-03
PA1609	fabB	beta-ketoacyl-ACP synthase I	-0.66	9.48E-03
PA3583	glpR	glycerol-3-phosphate regulon repressor	-0.66	1.18E-02

PA3255		hypothetical protein	-0.66	1.50E-02
PA2419		probable hydrolase	-0.66	1.13E-02
PA5306		conserved hypothetical protein	-0.66	1.25E-02
PA4345		hypothetical protein	-0.66	1.96E-03
PA4484	gatB	Glu-tRNA(Gln) amidotransferase subunit B	-0.67	1.52E-02
PA0901	aruE	N-Succinylglutamate desuccinylase	-0.67	2.51E-03
PA4748	tpiA	triosephosphate isomerase	-0.67	2.01E-03
PA2008	fahA	fumarylacetoacetase	-0.67	1.56E-03
PA4918	pcnA	nicotinamidase, PcnA	-0.67	3.48E-03
PA3273		hypothetical protein	-0.67	1.56E-02
PA3657	map	methionine aminopeptidase	-0.68	5.91E-03
PA3858		probable amino acid-binding protein	-0.68	5.76E-03
PA5080		prolyl aminopeptidase	-0.68	1.66E-02
PA3810	hscA	heat shock protein HscA	-0.68	2.37E-02
PA2619	infA	initiation factor	-0.68	4.37E-03
PA0024	hemF	coproporphyrinogen III oxidase, aerobic	-0.68	2.92E-02
PA0342	thyA	thymidylate synthase	-0.68	2.51E-02
PA3251		hypothetical protein	-0.68	4.60E-02
PA0296	spuI	Glutamylpolyamine synthetase	-0.69	8.05E-03
PA1900	phzB2	probable phenazine biosynthesis protein	-0.69	2.83E-02
PA4566	obg	GTP-binding protein Obg	-0.69	1.61E-02
PA3117	asd	aspartate semialdehyde dehydrogenase	-0.70	6.00E-03
PA4460	lptH	LptH	-0.70	1.27E-03
PA1806	fabI	NADH-dependent enoyl-ACP reductase	-0.70	2.28E-03
PA1794	glnS	glutaminyl-tRNA synthetase	-0.71	3.69E-03
PA3713	spdH	spermidine dehydrogenase, SpdH	-0.71	7.30E-03
PA0459		probable ClpA/B protease ATP binding subunit	-0.72	7.92E-03
PA2025	gor	glutathione reductase	-0.72	4.95E-03
PA4163		hypothetical protein	-0.72	6.93E-03
PA1946	rbsB	binding protein component precursor of ABC ribose transporter	-0.72	3.98E-03
PA5140	hisF1	imidazoleglycerol-phosphate synthase, cyclase subunit	-0.72	4.12E-02
PA2407	fpvC	FpvC	-0.72	5.21E-03
PA5111	gloA3	lactoylglutathione lyase	-0.72	3.76E-02
PA5396		hypothetical protein	-0.72	3.95E-03
PA4001	sltB1	soluble lytic transglycosylase B	-0.72	3.84E-02
PA4063		hypothetical protein	-0.72	5.33E-03
PA4769		probable transcriptional regulator	-0.72	1.59E-02
PA0775		conserved hypothetical protein	-0.72	3.58E-02
PA4069		hypothetical protein	-0.72	1.47E-02
PA4340		hypothetical protein	-0.72	1.59E-03
PA1596	htpG	heat shock protein HtpG	-0.73	3.39E-03
PA3977	hemL	glutamate-1-semialdehyde 2,1-aminomutase	-0.73	4.14E-03

PA3118	leuB	3-isopropylmalate dehydrogenase	-0.73	1.15E-02
PA5317	dppA5	probable binding protein component of ABC dipeptide transporter	-0.73	3.83E-03
PA3659		probable aminotransferase	-0.73	2.43E-02
PA5082	dguC	DguC	-0.73	7.93E-03
PA3083	pepN	aminopeptidase N	-0.74	1.23E-02
PA0002	dnaN	DNA polymerase III, beta chain	-0.74	1.35E-02
PA0085	hcp1	Hcp1	-0.74	5.75E-03
PA4275	nusG	transcription antitermination protein NusG	-0.74	3.35E-03
PA2044		hypothetical protein	-0.74	6.35E-03
PA3675		hypothetical protein	-0.74	4.63E-02
PA3005	nagZ	beta-N-acetyl-D-glucosaminidase	-0.75	1.75E-03
PA3162	rpsA	30S ribosomal protein S1	-0.76	4.13E-03
PA0546	metK	methionine adenosyltransferase	-0.76	9.16E-03
PA2699		hypothetical protein	-0.76	2.36E-03
PA4646	upp	uracil phosphoribosyltransferase	-0.76	2.52E-02
PA0446		conserved hypothetical protein	-0.76	3.98E-03
PA3751	purT	phosphoribosylglycinamide formyltransferase 2	-0.76	1.97E-02
PA1512	hcpA	secreted protein Hcp	-0.76	3.78E-03
PA2841		probable enoyl-CoA hydratase/isomerase	-0.76	9.88E-03
PA2007	maiA	maleylacetoacetate isomerase	-0.76	8.79E-03
PA0306.1		zed protein	-0.77	3.29E-02
PA5018	msrA	peptide methionine sulfoxide reductase	-0.77	2.14E-03
PA1205		conserved hypothetical protein	-0.77	9.19E-04
PA4587	ccpR	cytochrome c551 peroxidase precursor	-0.77	1.17E-02
PA5067	hisE	phosphoribosyl-ATP pyrophosphohydrolase	-0.77	4.87E-02
PA1349		conserved hypothetical protein	-0.77	1.17E-02
PA2968	fabD	malonyl-CoA-[acyl-carrier-protein] transacylase	-0.78	5.36E-04
PA5378	cbcX	CbcX	-0.78	1.00E-02
PA3582	glpK	glycerol kinase	-0.79	1.20E-02
PA0668	tyrZ	tyrosyl-tRNA synthetase 2	-0.79	1.24E-02
PA0381	thiG	thiamine biosynthesis protein, thiazole moiety	-0.79	1.58E-02
PA4389		3-oxacyl-acyl carrier protein reductase	-0.79	1.13E-03
PA4005		conserved hypothetical protein	-0.79	2.97E-02
PA4266	fusA1	elongation factor G	-0.80	6.03E-03
PA3635	eno	enolase	-0.80	2.27E-03
PA2442	gcvT2	glycine cleavage system protein T2	-0.80	7.90E-04
PA0482	glcB	malate synthase G	-0.80	2.14E-02
PA4267	rpsG	30S ribosomal protein S7	-0.80	2.35E-02
PA1796	folD	5,10-methylene-tetrahydrofolate dehydrogenase / cyclohydrolase	-0.80	7.32E-03
PA2531		probable aminotransferase	-0.80	2.11E-02
PA2970	rpmF	50S ribosomal protein L32	-0.80	3.88E-02
PA1327		probable protease	-0.81	6.64E-03

PA3139		probable amino acid aminotransferase	-0.81	3.53E-03
PA0972	tolB	TolB protein	-0.81	5.22E-03
PA0407	gshB	glutathione synthetase	-0.81	2.00E-03
PA1011		hypothetical protein	-0.81	3.59E-03
PA2410	fpvF	FpvF	-0.81	1.17E-04
PA1049	pdxH	pyridoxine 5'-phosphate oxidase	-0.81	5.87E-03
PA0654	speD	S-adenosylmethionine decarboxylase proenzyme	-0.81	1.62E-02
PA2850	ohr	organic hydroperoxide resistance protein	-0.81	3.33E-03
PA4316	sbcB	exodeoxyribonuclease I	-0.81	1.06E-02
PA1639		hypothetical protein	-0.81	1.89E-03
PA5076		probable binding protein component of ABC transporter	-0.82	1.21E-02
PA2575		hypothetical protein	-0.82	4.72E-03
PA3475	pheC	cyclohexadienyl dehydratase precursor	-0.82	2.81E-02
PA5481		hypothetical protein	-0.82	4.80E-02
PA2762		hypothetical protein	-0.82	2.63E-02
PA1781	nirB	assimilatory nitrite reductase large subunit	-0.82	1.45E-02
PA1772		probable methyltransferase	-0.83	1.11E-02
PA0793		hypothetical protein	-0.83	1.71E-02
PA0314		L-cysteine transporter of ABC system FliY	-0.83	7.33E-03
PA1618		conserved hypothetical protein	-0.83	1.44E-03
PA3427		probable short-chain dehydrogenases	-0.83	1.04E-03
PA1162	dapE	succinyl-diaminopimelate desuccinylase	-0.83	2.61E-02
PA0167		probable transcriptional regulator	-0.83	3.80E-02
PA4877		hypothetical protein	-0.83	1.69E-02
PA0301	spuE	polyamine transport protein	-0.84	7.11E-03
PA1677		conserved hypothetical protein	-0.84	3.99E-02
PA4645		probable purine/pyrimidine phosphoribosyl transferase	-0.84	8.57E-03
PA3818		extragenic suppressor protein SuhB	-0.84	5.99E-03
PA2040	pauA4	Glutamylpolyamine synthetase	-0.84	1.97E-02
PA1455	fliA	sigma factor FliA	-0.84	3.03E-02
PA1401		hypothetical protein	-0.84	8.69E-03
PA0895	aruC	N2-Succinylornithine 5-aminotransferase (SOAT) = N2-acetylornithine 5-aminotransferase (ACOAT)	-0.85	2.22E-02
PA1684		1,2-dihydroxy-3-keto-5-methylthiopentene dioxygenase MtnD	-0.85	2.02E-03
PA4569	ispB	octaprenyl-diphosphate synthase	-0.85	2.16E-04
PA0651	trpC	indole-3-glycerol-phosphate synthase	-0.85	5.69E-04
PA0019	def	polypeptide deformylase	-0.85	1.68E-02
PA2807		hypothetical protein	-0.85	2.85E-04
PA1337	ansB	glutaminase-asparaginase	-0.86	3.91E-03
PA5128	secB	secretion protein SecB	-0.86	6.84E-03
PA3208		conserved hypothetical protein	-0.86	1.71E-03
PA4708	phuT	Heme-transport protein, PhuT	-0.86	6.67E-03

PA4563	rpsT	30S ribosomal protein S20	-0.86	2.92E-03
PA2709	cysK	cysteine synthase A	-0.87	2.13E-03
PA0837	slyD	peptidyl-prolyl cis-trans isomerase SlyD	-0.87	1.25E-02
PA5066	hisI	phosphoribosyl-AMP cyclohydrolase	-0.87	4.99E-03
PA2856	tesA	lysophospholipase A	-0.87	5.23E-04
PA4255	rpmC	50S ribosomal protein L29	-0.87	4.44E-02
PA1624		hypothetical protein	-0.87	3.88E-02
PA4315	mvaT	transcriptional regulator MvaT, P16 subunit	-0.88	3.79E-02
PA4657		hypothetical protein	-0.88	6.53E-04
PA0449		hypothetical protein	-0.88	5.31E-03
PA5330		hypothetical protein	-0.88	4.19E-02
PA1562	acnA	aconitate hydratase 1	-0.89	1.98E-02
PA2071	fusA2	elongation factor G	-0.89	5.77E-03
PA4880		probable bacterioferritin	-0.89	5.62E-03
PA4920	nadE	NH3-dependent NAD synthetase	-0.89	1.67E-03
PA4434		probable oxidoreductase	-0.89	4.20E-03
PA2592		probable periplasmic spermidine/putrescine-binding protein	-0.89	1.40E-02
PA4384		hypothetical protein	-0.89	4.08E-03
PA3012		hypothetical protein	-0.89	1.30E-02
PA1748		probable enoyl-CoA hydratase/isomerase	-0.90	1.29E-02
PA5334	rph	ribonuclease PH	-0.90	2.44E-03
PA3724	lasB	elastase LasB	-0.90	1.63E-02
PA3488	tli5	Tli5	-0.90	3.38E-02
PA3627	ygbB	2C-methyl-D-erythritol 2,4-cyclodiphosphate synthase	-0.90	1.22E-02
PA1375	pdxB	erythronate-4-phosphate dehydrogenase	-0.90	7.43E-03
PA0283	sbp	sulfate-binding protein precursor	-0.90	4.24E-02
PA0590	apaH	bis(5'-nucleosyl)-tetraphosphatase	-0.90	1.90E-03
PA0795	prpC	citrate synthase 2	-0.91	6.60E-03
PA2950	fabV	FabV	-0.91	9.66E-04
PA5192	pckA	phosphoenolpyruvate carboxykinase	-0.91	4.28E-03
PA1965		hypothetical protein	-0.91	6.47E-03
PA2971		conserved hypothetical protein	-0.91	1.51E-02
PA1757	thrH	homoserine kinase	-0.92	1.01E-03
PA4308		conserved hypothetical protein	-0.92	3.79E-02
PA1166		hypothetical protein	-0.92	1.37E-03
PA0086	tagJ1	TagJ1	-0.92	2.40E-02
PA2764		hypothetical protein	-0.93	5.10E-03
PA1121	yfiR	YfiR	-0.93	6.60E-04
PA0036	trpB	tryptophan synthase beta chain	-0.94	3.01E-03
PA4031	ppa	inorganic pyrophosphatase	-0.94	3.08E-02
PA4015		conserved hypothetical protein	-0.94	2.26E-03
PA2851	efp	translation elongation factor P	-0.94	9.20E-03

PA3227	ppiA	peptidyl-prolyl cis-trans isomerase A	-0.94	2.85E-03
PA4458		conserved hypothetical protein	-0.94	1.60E-03
PA4747	secG	secretion protein SecG	-0.94	1.52E-02
PA3732		Uncharacterized protein	-0.94	5.49E-03
PA5489	dsbA	thiol:disulfide interchange protein DsbA	-0.95	7.16E-03
PA5190		NAD(P)H quinone oxidoreductase	-0.95	2.18E-03
PA2476	dsbG	thiol:disulfide interchange protein DsbG	-0.95	1.04E-02
PA2433		hypothetical protein	-0.96	1.22E-02
PA1151	imm2	pyocin S2 immunity protein	-0.96	4.48E-02
PA1023		probable short-chain dehydrogenase	-0.96	1.53E-02
PA3257	algO	AlgO	-0.97	1.05E-04
PA5285	sutA	SutA	-0.97	2.66E-02
PA0126		hypothetical protein	-0.97	3.06E-03
PA0653		conserved hypothetical protein	-0.97	2.37E-02
PA0706	cat	chloramphenicol acetyltransferase	-0.97	7.31E-03
PA0329		conserved hypothetical protein	-0.98	4.02E-04
PA5339		conserved hypothetical protein	-0.98	4.94E-03
PA0462		hypothetical protein	-0.98	5.25E-03
PA0542		conserved hypothetical protein	-0.98	2.16E-02
PA3155	wbpE	UDP-2-acetamido-2-dideoxy-d-ribo-hex-3-uluronic acid transaminase, wbpE	-0.99	7.68E-04
PA4406	lpxC	UDP-3-O-acyl-N-acetylglucosamine deacetylase	-0.99	1.61E-03
PA0594	surA	peptidyl-prolyl cis-trans isomerase SurA	-0.99	6.08E-04
PA3123		RidA subfamily protein	-1.00	2.27E-02
PA0309		hypothetical protein	-1.00	5.85E-03
PA4278		hypothetical protein	-1.00	1.97E-03
PA3302	phaJ1	conserved hypothetical protein	-1.01	7.86E-03
PA3169		5-methylthioribose-1-phosphate isomerase MtnA	-1.01	5.44E-04
PA0067	prlC	oligopeptidase A	-1.02	1.30E-02
PA4951	orn	oligoribonuclease	-1.02	2.51E-02
PA0937		conserved hypothetical protein	-1.02	5.31E-03
PA5425	purK	phosphoribosylaminoimidazole carboxylase	-1.02	3.98E-04
PA2204		probable binding protein component of ABC transporter	-1.02	1.99E-02
PA0964	pmpR	pqsR-mediated PQS regulator, PmpR	-1.02	3.71E-02
PA1605		hypothetical protein	-1.02	4.41E-02
PA2623	icd	isocitrate dehydrogenase	-1.03	1.21E-02
PA4043	ispA	geranyltranstransferase	-1.03	1.54E-02
PA1768		hypothetical protein	-1.03	6.87E-03
PA5137		hypothetical protein	-1.03	1.67E-03
PA5046		malic enzyme	-1.04	5.69E-03
PA5131	pgm	phosphoglycerate mutase	-1.04	7.62E-03
PA3647		probable outer membrane protein precursor	-1.04	2.81E-02

PA5138		hypothetical protein	-1.04	1.10E-03
PA3567		probable oxidoreductase	-1.04	2.32E-02
PA0905	rsmA	RsmA	-1.04	6.76E-03
PA3916	moaE	molybdopterin converting factor, large subunit	-1.04	6.96E-04
PA1260	lhpP	ABC transporter periplasmic-binding protein, LhpP	-1.05	9.75E-04
PA2394	pvdN	PvdN	-1.05	3.99E-03
PA0139	ahpC	alkyl hydroperoxide reductase subunit C	-1.06	5.96E-03
PA3618		conserved hypothetical protein	-1.06	3.88E-03
PA2562		hypothetical protein	-1.06	1.70E-02
PA2659		hypothetical protein	-1.06	7.94E-04
PA0884		probable C4-dicarboxylate-binding periplasmic protein	-1.06	1.54E-02
PA0888	aotJ	arginine/ornithine binding protein AotJ	-1.07	4.10E-03
PA2796	tal	transaldolase	-1.07	5.70E-03
PA2464		hypothetical protein	-1.07	1.56E-03
PA5162	rmlD	dTDP-4-dehydrorhamnose reductase	-1.08	6.70E-03
PA5472		hypothetical protein	-1.08	2.75E-02
PA0943		hypothetical protein	-1.09	3.81E-03
PA3956		hypothetical protein	-1.09	2.48E-02
PA2960	pilZ	type 4 fimbrial biogenesis protein PilZ	-1.10	2.60E-03
PA1155	nrdB	NrdB, tyrosyl radical-harboring component of class Ia ribonucleotide reductase	-1.11	7.44E-03
PA0355	pfpI	protease PfpI	-1.11	6.42E-03
PA4558		probable peptidyl-prolyl cis-trans isomerase, FkbP-type	-1.11	1.57E-02
PA0423	pasP	PasP	-1.12	2.45E-03
PA1793	ppiB	peptidyl-prolyl cis-trans isomerase B	-1.12	8.41E-03
PA2770		hypothetical protein	-1.12	3.71E-02
PA1533		conserved hypothetical protein	-1.12	3.37E-02
PA0935		conserved hypothetical protein	-1.12	8.76E-04
PA3807	ndk	nucleoside diphosphate kinase	-1.13	1.04E-02
PA3440		conserved hypothetical protein	-1.13	4.67E-02
PA0659		hypothetical protein	-1.13	1.06E-02
PA3698		hypothetical protein	-1.13	1.97E-03
PA3836		hypothetical protein	-1.15	2.86E-03
PA4252	rplX	50S ribosomal protein L24	-1.15	5.55E-04
PA4211	phzB1	probable phenazine biosynthesis protein	-1.15	1.62E-02
PA3003		hypothetical protein	-1.15	6.00E-04
PA3922		conserved hypothetical protein	-1.16	5.95E-03
PA1863	modA	molybdate-binding periplasmic protein precursor ModA	-1.16	4.77E-03
PA1008	bcp	bacterioferritin comigratory protein	-1.16	5.53E-04
PA0122	rahU	rahU	-1.16	2.69E-03
PA5275		conserved hypothetical protein	-1.16	2.75E-02
PA3655	tsf	elongation factor Ts	-1.17	8.40E-03

PA2952	etfB	electron transfer flavoprotein beta-subunit	-1.17	4.51E-03
PA2506		hypothetical protein	-1.17	3.72E-02
PA2614	lolA	lipoprotein localization protein LolA	-1.18	4.93E-03
PA1800	tig	trigger factor	-1.19	4.57E-03
PA5505		probable TonB-dependent receptor	-1.19	9.95E-04
PA0681	hxcT	HxcT	-1.19	4.70E-03
PA0950		probable arsenate reductase	-1.19	2.67E-02
PA4767		conserved hypothetical protein	-1.19	2.26E-02
PA4453		conserved hypothetical protein	-1.21	7.93E-03
PA3785		conserved hypothetical protein	-1.21	8.94E-03
PA1074	braC	branched-chain amino acid transport protein BraC	-1.22	5.26E-03
PA5217		probable binding protein component of ABC iron transporter	-1.22	2.97E-03
PA0660		NAD(P)H:quinone reductase	-1.23	4.67E-03
PA4500	dppA3	probable binding protein component of ABC transporter	-1.24	2.11E-03
PA2951	etfA	electron transfer flavoprotein alpha-subunit	-1.25	6.67E-03
PA4496	dppA1	probable binding protein component of ABC transporter	-1.25	2.64E-03
PA5288	glnK	nitrogen regulatory protein P-II 2	-1.26	2.80E-03
PA4913		probable binding protein component of ABC transporter	-1.26	2.03E-03
PA0579	rpsU	30S ribosomal protein S21	-1.27	1.29E-03
PA5133		conserved hypothetical protein	-1.28	7.86E-03
PA5359		hypothetical protein	-1.28	8.60E-03
PA5527		hypothetical protein	-1.28	2.15E-02
PA1112		conserved hypothetical protein	-1.29	3.43E-02
PA3653	frr	ribosome recycling factor	-1.30	1.54E-03
PA4755	greA	transcription elongation factor GreA	-1.30	1.09E-02
PA0852	cbpD	chitin-binding protein CbpD precursor	-1.31	3.78E-04
PA3621	fdxA	ferredoxin I	-1.32	7.69E-03
PA2184		conserved hypothetical protein	-1.32	1.24E-02
PA3332		conserved hypothetical protein	-1.33	1.73E-03
PA0300	spuD	polyamine transport protein	-1.34	9.98E-03
PA4366	sodB	superoxide dismutase	-1.35	7.27E-03
PA1342	aatJ	AatJ	-1.36	3.55E-03
PA1159		probable cold-shock protein	-1.36	1.58E-04
PA5153		amino acid (lysine/arginine/ornithine/histidine/octopine) ABC transporter periplasmic binding protein	-1.38	9.95E-03
PA1574		conserved hypothetical protein	-1.38	2.85E-03
PA2548		hypothetical protein	-1.39	5.15E-03
PA5167	dctP	DctP	-1.40	5.53E-03
PA0315		hypothetical protein	-1.40	4.33E-03
PA4739		conserved hypothetical protein	-1.40	4.13E-02
PA0604	agtB	AgtB	-1.40	9.33E-03
PA5148		conserved hypothetical protein	-1.41	3.34E-02

PA0552	pgk	phosphoglycerate kinase	-1.43	1.68E-03
PA3266	capB	cold acclimation protein B	-1.43	9.05E-04
PA1606		hypothetical protein	-1.44	2.40E-03
PA1579		hypothetical protein	-1.45	5.81E-03
PA0599		hypothetical protein	-1.45	1.23E-02
PA4393	ampG	AmpG	-1.46	4.72E-03
PA5305		conserved hypothetical protein	-1.47	3.69E-02
PA4716		hypothetical protein	-1.49	5.96E-03
PA3686	adk	adenylate kinase	-1.50	1.42E-03
PA1493	cysP	sulfate-binding protein of ABC transporter	-1.50	1.70E-03
PA3753		conserved hypothetical protein	-1.50	4.17E-03
PA3313		hypothetical protein	-1.53	7.67E-03
PA3250		hypothetical protein	-1.54	1.66E-02
PA0409	pilH	twitching motility protein PilH	-1.55	1.11E-02
PA4533		hypothetical protein	-1.56	6.99E-03
PA4847	accB	biotin carboxyl carrier protein (BCCP)	-1.57	5.10E-03
PA1830		hypothetical protein	-1.59	3.05E-02
PA3940		probable DNA binding protein	-1.61	1.14E-02
PA4922	azu	azurin precursor	-1.64	2.33E-03
PA5369	pstS	phosphate ABC transporter, periplasmic phosphate-binding protein, PstS	-1.65	3.93E-03
PA0971	tolA	TolA protein	-1.67	4.51E-05
PA3383		binding protein component of ABC phosphonate transporter	-1.68	3.86E-03
PA4611		hypothetical protein	-1.68	7.38E-03
PA0456		probable cold-shock protein	-1.69	5.56E-04
PA3796		hypothetical protein	-1.76	1.34E-02
PA0059	osmC	osmotically inducible protein OsmC	-1.82	1.24E-02
PA5052		hypothetical protein	-1.82	2.23E-04
PA1358		hypothetical protein	-1.84	1.41E-02
PA0039		hypothetical protein	-1.85	5.46E-03
PA3351	flgM	FlgM	-1.88	2.71E-02
PA2318		hypothetical protein	-1.91	4.70E-04
PA2146		conserved hypothetical protein	-1.98	2.22E-02
PA1480	ccmF	cytochrome C-type biogenesis protein CcmF	-2.05	1.99E-03

Table S2. Detected metabolites that exhibit abundance differences between KM-5-35-treated *P. aeruginosa* cells and untreated control.

+/-	m/z	Metabolite	Log_2FC (treated /unt)	Score	*p* value	Mass Error (ppm)	Database*
+	136.0631	Adenine	-0.8	47	1.01E-01	9.84	*b*
+	348.07129	Adenosine monophosphate	-0.65	44.6	1.05E-01	2.67	*b*
-	426.0214	ADP	-0.38	48.2	1.94E-01	-1.74	*b*
+	330.06003	cAMP	0.29	45.2	3.90E-01	0.7	*b*
-	191.01855	Citric acid	-1.32	40.4	2.36E-04	-6.13	*b*
-	402.00794	Cytidine diphosphate (CDP)	0.94	40.3	4.20E-03	-7.35	*b*
-	481.97762	Cytidine triphosphate (CTP)	0.96	42.5	2.05E-02	0.8	*b*
+	259.02072	D-Glucose 6-phosphate	-0.53	46.8	2.26E-01	-6.59	*a*
-	168.98915	D-Glyceraldehyde 3-phosphate	-0.42	43.6	2.33E-01	-9.43	*b*
+	106.05056	D-Serine	0.32	45.7	3.43E-01	6.55	*b*
+	428.03726	Deoxyguanosine diphosphate (dGDP)	-1.29	40.3	1.02E-02	1.34	*b*
+	195.00346	DL-Glycerol 1-phosphate	0.3	41.6	2.90E-01	3.28	*b*
-	321.04805	dTMP	0.08	42.6	8.78E-01	-3.95	*b*
+	786.16427	Flavin adenine dinucleotide (FAD)	-0.19	46.7	2.20E-01	-0.18	*b*
-	455.09741	Flavine mononucleotide (FMN)	0.26	40.8	4.28E-01	0.16	*b*
-	306.07523	Glutathione	-2.15	44.4	3.26E-02	-4.24	*b*
-	611.14263	Glutathione, oxidized	-1.88	42.7	1.02E-02	-3.35	*b*
+	364.06613	Guanosine monophosphate	-1.01	48	2.55E-02	2.34	*b*
+	152.05674	Guanine	-0.89	49.2	2.82E-02	0.33	*b*
+	444.03207	Guanosine 5'-diphosphate (GDP)	-1.83	41.1	2.74E-02	1.05	*b*
+	90.05573	L-Alanine	-1.17	42.3	2.46E-04	8.71	*a*
+	134.0456	L-Aspartic acid	-0.98	41.2	1.38E-02	6.16	*a*
+	122.02745	L-Cysteine	-2.7	51.7	1.31E-02	3.48	*a*
+	148.06099	L-Glutamic acid	-2.86	45.6	1.75E-03	3.78	*a*
+	156.07715	L-Histidine	0.19	55.9	5.40E-01	2.59	*a*
+	147.11365	L-Lysine	-0.39	45.8	2.04E-01	5.8	*a*
+	150.05868	L-Methionine	-0.48	42.3	1.94E-02	2.38	*a*
+	166.08643	L-Phenylalanine	1.54	48.5	7.54E-05	1.06	*a*
+	116.07143	L-Proline	0.89	46.2	5.19E-05	7.13	*a*
+	106.05056	L-Serine	-0.88	41.7	5.09E-02	6.59	*a*
+	205.09703	L-Tryptophan	1.22	46.4	8.53E-04	-0.63	*b*
+	118.08698	L-Valine	3.02	50.5	2.23E-02	6.19	*a*
-	664.1157	NADH	-1.47	40.1	3.26E-02	-2.7	*b*
-	284.12364	Nicotianamine	-3	40.7	4.69E-02	-5.11	*c*
-	146.04467	O-Acetyl-L-serine	-3.04	40.6	3.92E-03	-8.21	*b*

+	249.0634	Pyridoxamine-5'-Phosphate	-1.33	43.4	2.25E-02	-0.33	*b*
-	117.0182	Succinic acid	-0.93	51.5	5.20E-02	-9.15	*a*
-	402.99423	Uridine diphosphate (UDP)	-0.69	49.4	3.56E-02	-1.72	*b*
-	323.02802	Uridine monophosphate (UMP)	-0.95	51.8	2.16E-02	-1.75	*b*

*Metabolites were identified using the Progenesis QI untargeted metabolomics workflow including LC-MS/MS alignment, feature detection, normalization, and database matching against in-house-built MS/MS libraries, public MS/MS libraries, and structure libraries coupled to theoretical fragmentation. The in-house MSF database contains reference standards with experimentally validated retention time and fragmentation profiles, while additional annotations were assigned using public spectral and structure databases based on accurate mass, isotopic pattern, and fragmentation similarity.

(a) identified using the in-house mass spectrometry facility (MSF) reference database with authentic standards.

(b) identified using METLIN MS/MS library

(c) identified using HMDB structure databases and theoretical fragmentation.

Table S3. Quorum sensing proteins detected in KM-5-35-treated *P. aeruginosa* cells relative to untreated control

Locus ID	Name	Log_2FC (treated/unt)	p value
PA0996	PqsA	0.29	2.98E-02
PA0997	PqsB	0.25	2.1.E-01
PA0998	PqsC	-0.09	5.59E-01
PA0999	PqsD	0.36	3.50E-02
PA1000	PqsE	0.42	2.31E-02
PA3476	RhlI	1.44	1.67E-01
PA3478	RhlB	0.81	1.02E-01
PA3479	RhlA	0.73	4.12E-02
PA1871	LasA	ND	-
PA3724	LasB	-0.9	1.63E-02
PA0051	phzH	0.74	2.82E-02
PA1899	PhzA2	0.68	4.93E-02
PA1900	PhzB2	-0.69	2.83E-02
PA1904	PhzF2	0.32	8.58E-01
PA4209	PhzM	0.58	4.65E-03
PA4211	PhzB1	-1.15	1.62E-02
PA4212	PhzC1	1.03	1.52E-02
PA4213	PhzD1	0.37	2.10E-02
PA4214	PhzE1	0.45	5.97E-02
PA4217	PhzS	-0.55	1.22E-02

Table S4. Efflux pumps detected in KM-5-35-treated *P. aeruginosa* cells relative to untreated control

Locus ID	Name	Log_2FC (treated/unt)	*p* value
PA0156	TriA	0.66	2.33E-03
PA0157	TriB	0.48	1.08E-02
PA0158	TriC	0.71	1.76E-03
PA0425	MexA	0.65	2.57E-03
PA0426	MexB	0.78	6.57E-04
PA0427	OprM	-0.22	5.30E-01
PA2019	MexX	0.82	5.71E-02
PA2020	MexZ	1.03	1.12E-02
PA2491	MexS	-0.18	7.32E-01
PA2492	MexT	0.37	1.13E-03
PA2493	MexE	0.81	3.88E-04
PA2494	MexF	0.58	1.73E-02
PA2495	OprN	0.61	6.51E-03
PA4374	MexV	0.57	1.27E-02
PA4375	MexW	0.28	1.93E-02
